# Inhibition of USP28 overcomes Cisplatin-resistance of squamous tumors by suppression of the Fanconi anemia pathway

**DOI:** 10.1038/s41418-021-00875-z

**Published:** 2021-10-05

**Authors:** Cristian Prieto-Garcia, Oliver Hartmann, Michaela Reissland, Thomas Fischer, Carina R. Maier, Mathias Rosenfeldt, Christina Schülein-Völk, Kevin Klann, Reinhard Kalb, Ivan Dikic, Christian Münch, Markus E. Diefenbacher

**Affiliations:** 1 Protein Stability and Cancer Group, Department of Biochemistry and Molecular Biology, University of Würzburg, Würzburg, Germany; 2 Comprehensive Cancer Centre Mainfranken, Würzburg, Germany; 3 Department for Radiotherapy, University Hospital Würzburg, Würzburg, Germany; 4 Department of Biochemistry and Molecular Biology, Würzburg, Germany; 5 Institute for Pathology, University of Würzburg, Würzburg, Germany; 6 Core Unit High-Content Microscopy, Biocenter, University of Würzburg, Würzburg, Germany; 7 Protein Quality Control Group, Institute of Biochemistry II, Goethe University Frankfurt, Frankfurt, Germany; 8 Institute for Human Genetics, Biocenter, University of Würzburg, Würzburg, Germany; 9 Molecular Signaling Group, Institute of Biochemistry II, Goethe University Frankfurt, Frankfurt, Germany

**Keywords:** Cancer genetics, Cancer, Experimental models of disease

## Abstract

Squamous cell carcinomas (SCC) frequently have an exceptionally high mutational burden. As consequence, they rapidly develop resistance to platinum-based chemotherapy and overall survival is limited. Novel therapeutic strategies are therefore urgently required. SCC express ∆Np63, which regulates the Fanconi Anemia (FA) DNA-damage response in cancer cells, thereby contributing to chemotherapy-resistance. Here we report that the deubiquitylase USP28 is recruited to sites of DNA damage in cisplatin-treated cells. ATR phosphorylates USP28 and increases its enzymatic activity. This phosphorylation event is required to positively regulate the DNA damage repair in SCC by stabilizing ∆Np63. Knock-down or inhibition of USP28 by a specific inhibitor weakens the ability of SCC to cope with DNA damage during platin-based chemotherapy. Hence, our study presents a novel mechanism by which ∆Np63 expressing SCC can be targeted to overcome chemotherapy resistance. Limited treatment options and low response rates to chemotherapy are particularly common in patients with squamous cancer. The SCC specific transcription factor ∆Np63 enhances the expression of Fanconi Anemia genes, thereby contributing to recombinational DNA repair and Cisplatin resistance. Targeting the USP28-∆Np63 axis in SCC tones down this DNA damage response pathways, thereby sensitizing SCC cells to cisplatin treatment.

## Introduction

Squamous tumors arise in multiple tissues and are among the most highly mutated cancer entities [1–3]. Current treatment options are DNA damage-inducing chemotherapy or personalized approaches, such as receptor tyrosine kinases (RTK) and immune checkpoint inhibitors [4–10]. Although tumours initially respond, they frequently develop resistance [11, 12]. One possible strategy to prevent the ability of cancer cells to escape treatment is the targeting of proteins involved in maintaining SCC identity or survival. One such factor, which distinguishes SCC from other tumour entities, is ∆Np63, a proto-oncogenic transcription factor and member of the TP53 superfamily that is a the master regulator of SCC formation [13–15].

SCC tumors depend on ∆Np63 to maintain basal epithelial cell identity [16–19]. Acute depletion of ∆Np63 in an autochthonous SCC model results in rapid tumor regression [20]. This is in part mediated by direct interference with TAp73-dependent apoptosis as witnessed by enhanced expression of the pro-apoptotic genes PUMA and NOXA [21]. Furthermore, ∆Np63 contributes to the resistance of SCC towards platin-based chemotherapy due to its ability to regulate the expression of DNA-damage response (DDR) genes [22]. Specifically, genes encoding proteins of the Fanconi Anemia pathway are directly regulated by ∆Np63 [23]. ∆Np63 binds to the promoters of several genes of this pathway and drives their expression during therapy [23].

A vulnerability of SCC is its dependence on the deubiquitylase (DUB) USP28 [24]. USP28 stabilizes the ∆Np63 protein and is required for SCC tumor maintenance. Consistently, a first-generation small molecule inhibitor of USP28 (AZ1) suppressed tumor growth in a murine isogenic transplant model [24]. Via several of its substrates, USP28 is involved in chromatin stability, segregation and DNA damage signaling, but whether it has a role in the response to DNA damage is unclear [25–27]. Here we report that USP28 maintains via ∆Np63 the genomic integrity of SCCs during chemotherapy with cisplatin and that targeting USP28 sensitizes ∆Np63 positive SCC to chemotherapy.

## Results

### USP28 is recruited to sites of DNA damage and phosphorylated by ATR, upon Cisplatin treatment

**Fig. 1:**
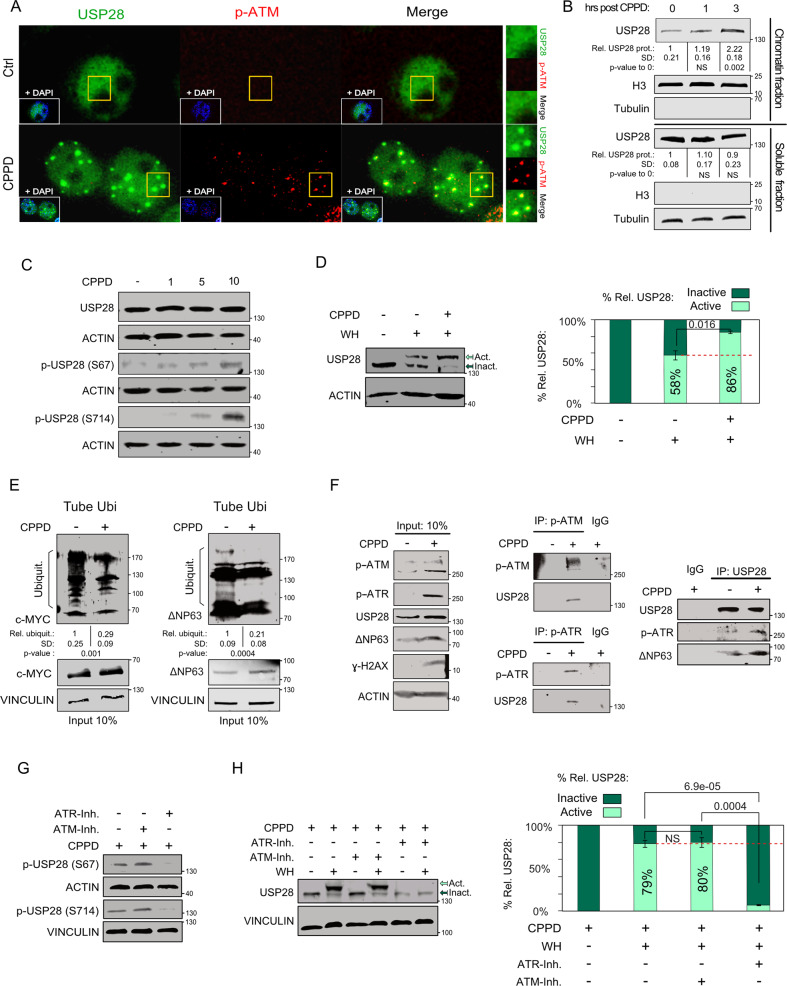
USP28 is recruited to DNA damage sites and phosphorylated by ATR upon Cisplatin treatment. **A** Immunofluorescence staining of endogenous USP28 and phospho-ATM in A431 cells exposed to either DMF or 5 µM Cisplatin for 6 h. DAPI served as nuclear counterstain. Scale bar = 10 μm. *n* = 3. **B** Chromatin and nucleoplasm fractionation, followed by immunoblotting of endogenous USP28 in A431 cells exposed to 5 µM CPPD for indicated time points. Histone H3 and TUBULIN serve as loading and fractionation control. Relative amount of protein abundance and standard deviation (SD) are calculated from three independent biological replicates. Relative protein intensities were quantified respect to sample “0 h post CPPD” upon H3 or TUBULIN normalization. *p* values were calculated using two‐tailed *T* test statistical analysis. Representative immunoblots of *n* = 3. **C** Immunoblotting of total and phosphorylated USP28 at serine 67 and 714 in A431 cells exposed to indicated concentrations of CPPD for 6 h. Representative immunoblots of *n* = 3. **D** Ubiquitin suicide probe (warhead) assay, followed by immunoblotting against USP28 in A431 cells exposed to 5 µM CPPD for 6 h. “Act.” arrow indicates active USP28. “Inact.” arrow indicates inactive USP28. ACTIN serves as loading control. Bar graph shows quantification of active and inactive USP28 upon ACTIN normalization. Quantitative graphic is represented as mean and standard deviation (SD) of three independent biological replicates. *p* values were calculated using two‐tailed *T* test statistical analysis. **E** Tandem-ubiquitin binding entity (TUBE) pulldown of endogenous ubiquitin, followed by immunoblotting against endogenous c-MYC and ∆Np63 in control (DMF) or 5 µM CPPD treated A431 cells for 6 h. VINCULIN serves as loading control. Relative ubiquitylated protein abundance and standard deviation of *n* = 3 experiments were quantified. *p* values were calculated using two‐tailed *T* test statistical analysis. *n* = 3. **F** Immunoblot of A431 cells treated with either control (DMF) or 5 µM CPPD for 6 h, followed by immunoprecipitation of control rabbit IgG, endogenous phospho-ATM (ser1981), phospho-ATR (ser428) or USP28 antibody and consecutive immune-blotting against ATM, ATR, USP28, ɣ-H2AX or ∆Np63 with specific antibodies, respectively. ACTIN serves as loading control. Representative immunoblots of *n* = 3 independent experiments. **G** Immunoblotting of phosphorylated USP28 at serine 67 and 714 in A431 cells exposed to 5 µM CPPD for 6 h and co-treatment with 15 µM ATM kinase inhibitor KU55933 (ATM inh.) or 2.5 µM VE 821 (ATR inh.). ACTIN and VINCULIN serve as loading control. Representative immunoblots of *n* = 3. **H** Ubiquitin suicide probe (warhead) assay, followed by immunoblotting against USP28 in A431 cells exposed to 5 µM CPPD for 6 h and/or co-treatment with 15 µM ATM kinase inhibitor KU55933 or 2.5 µM ATR kinase inhibitor VE 821. Act. arrow indicates active USP28. Inact. arrow indicates inactive USP28. VINCULIN serves as loading control. Bar graph shows quantifications of active and inactive USP28 upon VINCULIN normalization. Quantitative graphic is represented as mean and standard deviation (SD) of three independent biological replicates (*n* = 3). *p* values were calculated using two‐tailed *T* test statistical analysis. See also Fig. S1.

Previous studies demonstrated that USP28 is recruited to DNA damage sites upon ionizing radiation [26]. To test whether DNA crosslinking agents induce a similar response, we exposed the human SCC line A431 to either DMF or Cisplatin (CPPD), followed by immunofluorescence staining against USP28 and phospho-ATM (Figs. 1A and S1A). Exposure to CPPD resulted in re-localization of USP28 to distinct nuclear foci and colocalization with the activated, hence phosphorylated, DNA damage kinase ATM (Figs. 1A and S1B). Also, the fraction of USP28 that associates with chromatin increased in a time-dependent fashion after CPPD treatment (Fig. 1B). We wondered if USP28 is a substrate for ATM/ATR mediated phosphorylation. USP28 harbours two SQ/TQ motifs at serines 67 and 714 (Fig. S1C, D) and was phosphorylated in a CPPD-dependent manner at both sites (Fig. 1C). This was accompanied by an increase in its enzymatic activity (Fig. 1C, D) and an enhanced deubiquitylation of USP28 substrates (Fig. 1E). Conversely, USP28 knock-down reduced the levels of ∆Np63, c-MYC and c-JUN after CPPD treatment (Fig. S1E). Stabilization of these onco-proteins during CPPD exposure indeed depends on USP28, as the lung cancer cell line SK-MES1 (SCC), which is mutant for USP28 and expresses low levels thereof, showed some degree of c-JUN or c-MYC destabilization after CPPD treatment (Fig. S1F–H). Activation of the DNA damage response, however, was not affected as indicated by increased levels of ɣ-H2AX (Fig. S1H).

We next asked whether ATM or ATR phosphorylates USP28 upon CPPD treatment. ATR is frequently upregulated or amplified in SCC compared to ADC, while ATM is commonly downregulated or lost, suggesting that SCC predominantly rely on ATR to activate the DDR cascade (Fig. S1I, J). Immunoprecipitation of auto-phosphorylated ATM or ATR coprecipitated USP28 in CPPD-treated A431 cells (Fig. 1F). Conversely, endogenous USP28 co-immunoprecipitated phosphorylated ATR and ∆Np63 (Fig. 1F). Notably, binding of p-ATR and ∆Np63 to USP28 was increased after CPPD treatment (Fig. 1F). Inhibition of ATR via VE-821, but not ATM using KU55933, decreased the phosphorylation of USP28 and strongly reduced the DUB activity of USP28, resulting in an overall reduction of USP28 abundance (Fig. 1G, H). Our data show that USP28 is phosphorylated in an ATR-dependent manner upon CPPD treatment and argue that phosphorylated USP28 shows higher deubiquitylase activity.

### Phosphorylation of USP28 upon Cisplatin exposure is required to repair DNA damage in SCC

**Fig. 2:**
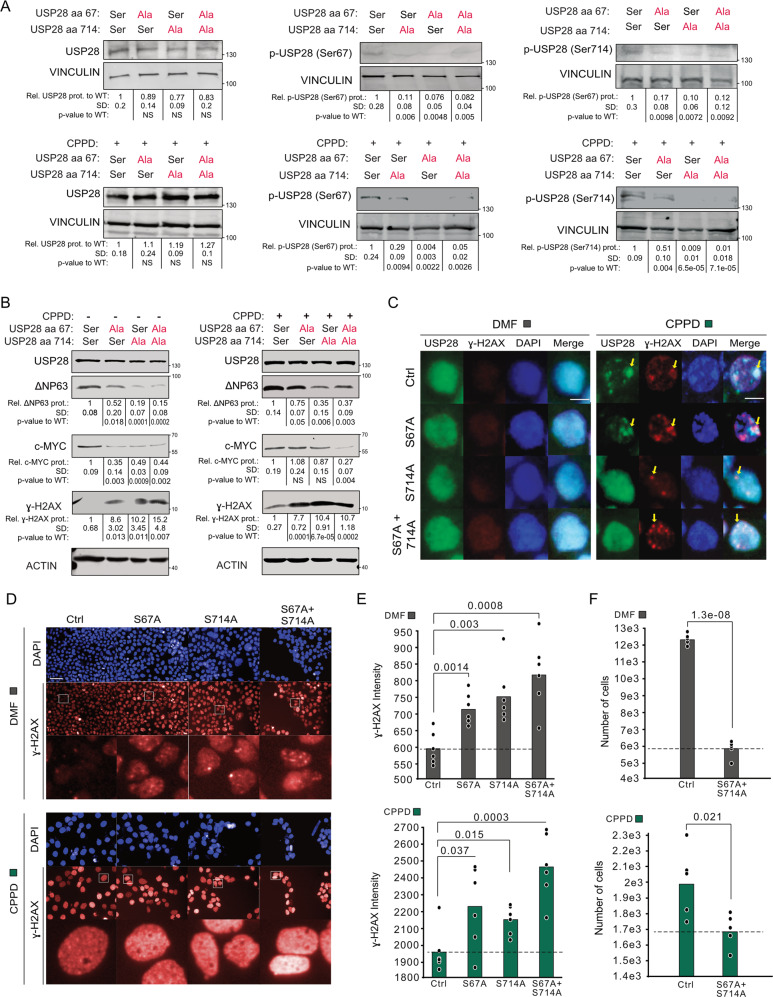
Phosphorylation of USP28 upon Cisplatin exposure is required to repair DNA damage in SCC. **A** Immunoblotting against endogenous USP28 and phosphorylated USP28 at serine 67 and 714 in control, S67A, S714A and compound S67A + S714A mutant A431 cells treated with either solvent control (DMF) or 5 µM CPPD for 6 h. VINCULIN serves as loading control. Representative immunoblots of *n* = 3. Relative amount of protein abundance and standard deviation (SD) are calculated from three independent biological replicates. Relative protein intensities were quantified respect to USP28 non-mutated cells upon VINCULIN normalization. *p* values were calculated using two‐tailed *T* test statistical analysis. **B** Immunoblotting of USP28, c-MYC, ∆Np63 and ɣ-H2AX in control, S67A, S714A and compound S67A + S714A mutant A431 cells treated with either solvent control (DMF) or 5 µM CPPD for 6 h. ACTIN serves as loading control. Representative immunoblots of *n* = 3. Relative amount of protein abundance and standard deviation (SD) are presented from *n* = 3 experiments. Relative protein intensities were quantified respect to USP28 non-mutated cells upon ACTIN normalization. *p* values were calculated using two‐tailed *T* test statistical analysis. **C** Immunofluorescence against endogenous USP28 and ɣ-H2AX in control, S67A, S714A and compound S67A + S714A mutant A431 cells treated with either solvent control (DMF) or 5 µM CPPD for 6 h. Scale bar = 10 μm. DAPI served as nuclear marker. *n* = 3. **D** Immunofluorescence against endogenous phosho-H2AX in control, S67A, S714A and compound S67A + S714A mutant A431 cells treated with either DMF (gray) or 5 µM CPPD (green) for 24 h. DAPI served as nuclear marker. *n* = 6. **E** Quantification of (**D**). DMF (gray) or 5 µM CPPD (green) for 24 h. ɣ-H2AX intensity was calculated measuring 15 fields per well (*n* = 6). Quantitative graphic is represented as mean of six independent biological replicates (black dots). Scale bar = 100 μm. *p* values were calculated using two‐tailed *T* test statistical analysis. **F** Quantification of (**D**). Number of cells in control and S67A + S714A mutant A431 cells, treated with either DMF (gray) or 5 µM CPPD (green) for 24 h. Number of cells were calculated measuring 15 fields per well (*n* = 5). Quantitative graphic is represented as mean of five independent biological replicates (black dots). *p* values were calculated using two‐tailed *T* test statistical analysis. See also Fig. S2.

To examine the role of USP28 in the response to cisplatin, we generated knock-in cell lines for serine 67 (S67A), serine 714 (S714A), or both (S67/714 A) using CRISPR/Cas9 (Fig. S2A). All mutant cell lines showed nearly wildtype levels of total USP28 but no phosphorylated USP28 using antibodies that recognize the respective phosphorylated site for S67A and S714A, as expected (Fig. 2A). Upon exposure to CPPD, USP28 was phosphorylated at reduced levels in the knock-in cells compared to wildtype. The faint band might represent low levels of wildtype cells after selection. Mutation of S714 and S67/714 A was associated with a decrease in the USP28 deubiquitylase activity upon exposure to CPPD (Fig. S2B, C). Similar results were obtained in the human cell line HEK-293T (Fig. S2D, E).

Mutations of the SQ/TQ motifs led to minor changes in the protein levels of c-MYC and ∆NP63 (Fig. 2B) in USP28^S67A^ cells, but resulted in a strong decline of both substrates in USP28^S714A^ or USP28^S67/714A^ cells. All mutant knock-in cell lines showed increased levels of phospho-TP53 (S15) and ɣ-H2AX under normal culture conditions and after CPPD treatment (Figs. 2B and S2F). Immunofluorescence stainings showed that USP28 was evenly distributed throughout the nucleus in control cells; after CPPD treatment, control and USP28^S67A^ cells displayed USP28 foci, which partially overlapped with ɣ-H2AX foci (Fig. 2C). In contrast, neither USP28^S714A^ or USP28^S67/714A^ showed CPPD-induced relocation of USP28, although ɣ-H2AX foci were detectable in wild type and mutant cells upon exposure to CPPD (Fig. 2C).

Mutation of the SQ/TQ motifs within USP28 induced basal replication and DDR stress as witnessed by increased ɣ-H2AX abundance under control conditions (Fig. 2D, E). Upon treatment with CPPD, mutant cells showed a significant increase in the number of ɣ-H2AX foci (Fig. 2D, E). Similar effects were seen with the DNA damage marker TP53BP1 in mutant A431 cells under basal and CPPD-treated conditions (Fig. S2H, I). It is noteworthy that mutation of the ATM/ATR phospho-sites within USP28 could affect DNA integrity under basal conditions [28]. In our experiments, the total number of cells was significantly reduced when cells carried mutations at S714A or S67/714 A (Figs. 2F and S2G) and showed increased replication stress as indicated by ɣ-H2AX and TP53BP1 foci.

### Loss of USP28 negatively affects the expression of DDR effector proteins in SCC

**Fig. 3:**
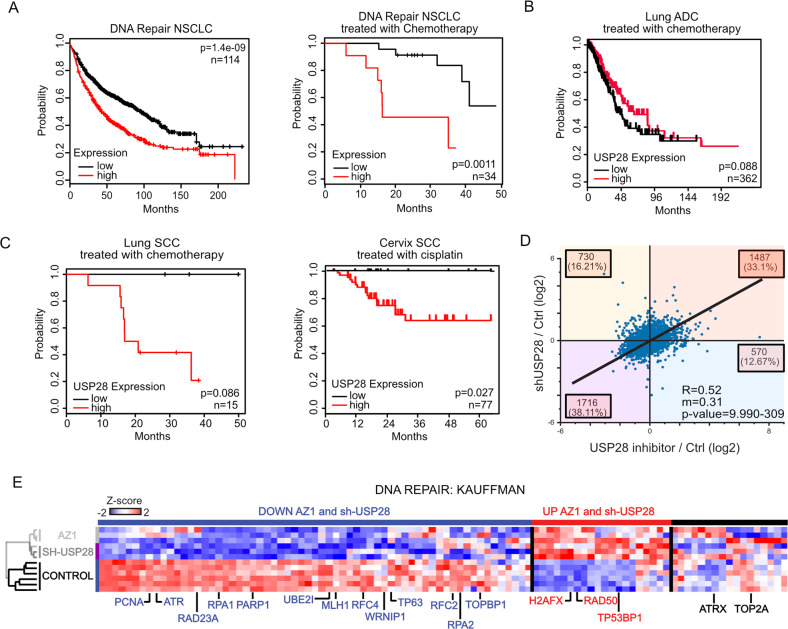
Loss of USP28 negatively affects the expression of DDR effector proteins in SCC. **A** Public available patient survival data of NSCLC patients are stratified by relative expression of DNA damage gene expression according to Kauffmann signature selection. Left panel= All NSCLC patients; Right panel= Only NSCLC treated with chemotherapy. *n* = 114 and *n* = 34, respectively. Generated with the open source tool www.kmplot.com. **B** Public available patient survival data of lung ADC patients treated with chemotherapy and stratified by relative expression of USP28. *n* = 362. Generated with the online tools www.kmplot.com and www.r2.amc.nl. **C** Publicly available patient survival data of lung and cervix SCC cancer patients treated with cisplatin (CPPD) and stratified by relative expression of USP28. Lung SCC *n* = 15 and Cervix SCC *n* = 77. Generated with the online tool www.r2.amc.nl. **D** Correlation of protein changes upon transduction of A431 cells with an inducible shRNA targeting USP28 (sh-USP28) relative to non-targeting control (Ctrl) and A431 cells treated with the DUB inhibitor AZ-1 relative to cells treated to DMSO (Ctrl). *n* = 3. Sh-USP28 and sh-NTC cells were exposed to 1 µg/ml Doxycycline for 72 h prior to analysis. A431 cells were exposed to AZ1 for 48 h prior to analysis. The diagonal line reflects a regression build on a linear model. R: Spearman’s correlation coefficient, m: slope of the linear regression model. Total proteins analysed = 4503. **E** Heatmap analysis according to the Kauffmann DNA damage protein signature of A431 cells upon exposure to AZ-1 (15 µM, 48 h) or DMSO (control) and the transduction of A431 cells with an inducible shRNA targeting USP28 (sh-USP28) or non-targeting control (Control). Sh-USP28 and sh-NTC cells were exposed to 1 µg/ml Doxycycline for 72 h prior to analysis. A431 cells were exposed to 15 µM AZ1 for 48 h prior to analysis. Blue = Down-regulated in AZ1/sh-USP28; Red = Up-regulated in AZ1/sh-USP28. *n* = 3. See also Fig. S3.

SCC tumors exhibit limited response to therapy and consequently poorer prognosis than ADC in overall survival [24, 29]. Interestingly, multiple DDR genes are expressed at significantly higher levels in lung SCC than in normal tissues and ADCs (Fig. S3A). A direct comparison of gene expression signatures of SCCs and ADCs shows that DDR gene expression is correlated with poor prognosis in SCCs (Figs. 3A and S3B).

Interestingly, high levels of USP28 are correlated with resistance to chemotherapy in oesophageal- and lung squamous cancer (Fig. S3C) [30–32]. Therefore, we stratified patient survival datasets for lung ADC and SCC regarding USP28 expression. A strong correlation of USP28 expression and overall survival was found for SCCs, but not for ADCs (Fig. 3B, C). Similar observations were obtained by analyzing publicly available datasets of cervix SCC upon cisplatin treatment (Fig. 3C). Patients with USP28^high^ tumors had a significantly lower probability to survive upon chemotherapy than patients assigned to the USP28^low^ cohort.

To test whether USP28 maintains expression of SCC signature genes, we used doxycycline-inducible shRNAs targeting USP28 and treated cells with the USP25/28 inhibitor AZ1 [33]. Silencing or AZ1-mediated inhibition of USP28 led to similar expression changes as measured by whole proteome mass spectrometry, with predominant effects on pathways associated with cellular stress, cell cycle progression and DNA damage checkpoint and repair (Figs. 3D, E and S3D). Our data indicate an upregulation of DNA damage sensors like TP53BP1, MRE11 or RAD50 and simultaneously the downregulation of proteins involved in replication-coupled DNA repair, such as RAD51, RPA1 or RPA2 (Figs. 3E and S3F). Of note, loss of USP28 activity was associated with a significant decrease of proteins involved in DNA replication (Fig. 3E).

### USP28-∆Np63 axis is required for chemoresistance in SCC

**Fig. 4:**
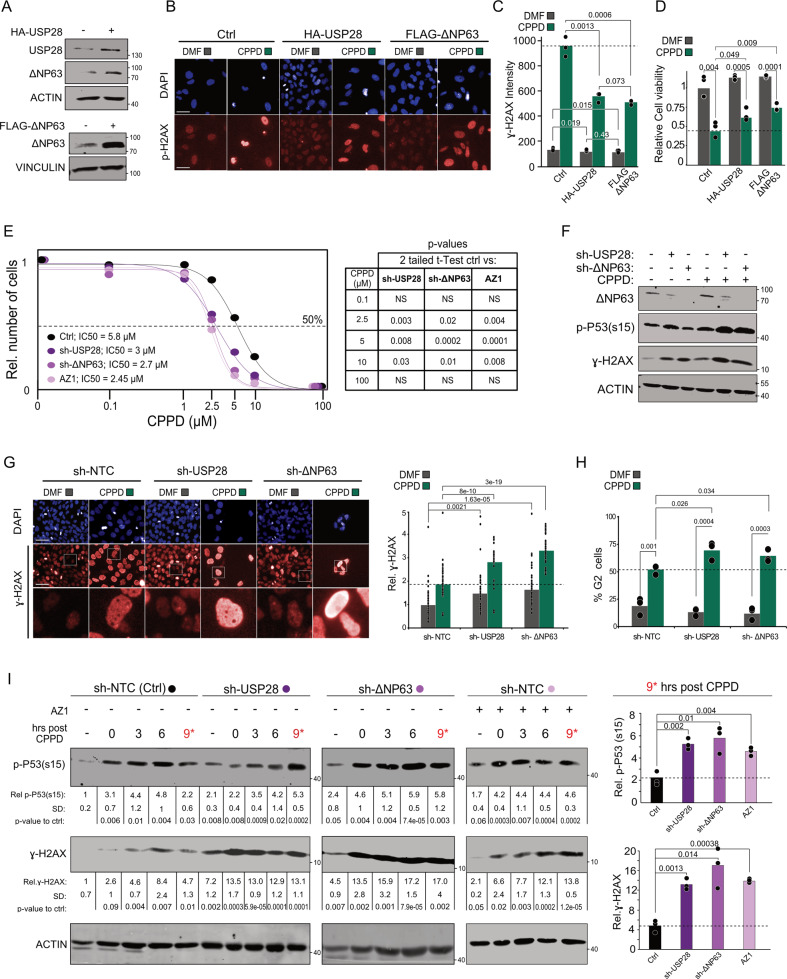
USP28-∆Np63 axis is required for DDR upon cisplatin treatment and chemoresistance in SCC. **A** Immunoblotting of USP28 and ∆Np63 in BEAS-2B cells transiently transfected with either human USP28 or ∆Np63. Transfection of a GFP cDNA expressing plasmid served as control (−). ACTIN and VINCULIN served as loading control. *n* = 3. **B** Immunofluorescence staining against the DNA damage marker ɣ-H2AX in BEAS-2B cells transiently transfected with constructs from (**A**) and exposed to 2.5 µM CPPD or DMF (Ctrl) for 48 h. Transfection of a GFP cDNA expressing plasmid served as control (Ctrl). DAPI served as nuclear marker. Highlighted images from Fig. S4A. *n* = 3. Scale bar = 100 μm. **C** Quantification of relative ɣ-H2AX fluorescence intensity in BEAS-2B from (**B**). 15 fields per well (*n* = 3) were quantified. Quantitative graphic is represented as mean of three independent biological replicates (black dots). *p* values were calculated using two‐tailed *T* test statistical analysis. **D** Quantification of relative cell survival in BEAS-2B from (**B**). 15 fields per well (*n* = 3) were quantified. Quantitative graphic is represented as mean of three independent biological replicates (black dots). *p* values were calculated using two‐tailed *T* test statistical analysis. **E** Quantification of relative cell survival (IC_50_) of A431 cells, either after shRNA knock down of USP28 (sh-USP28#1) or ∆NP63 (sh-∆NP63#1), or treated with 15 µM AZ-1, with co-exposure to either DMF (solvent control) or increasing concentrations of CPPD (0.1, 2.5, 5, 10, and 100 µM) for 48 h. *n* = 3. Quantitative plot is represented as mean of three independent biological replicates. *p* values were calculated using two‐tailed *T* test statistical analysis. **F** Immunoblot of endogenous ∆NP63, phospho-serine 15 TP53, ɣ-H2AX in lentivirally transduced A431 cells (shRNA-NTC, shRNA USP28#1 or ∆NP63#1), exposed to DMF or 5 µM CPPD for 24 h. ACTIN served as loading control. Representative immunoblot of *n* = 3. **G** Immunofluorescence staining against ɣ-H2AX in lentivirally transduced A431 cells (shRNA-control, shRNA USP28#1 or ∆NP63#1) upon exposure to either DMF or 5 µM CPPD for 48 h. DAPI served as nuclear marker. Quantification of relative ɣ-H2AX fluorescence intensity in A431 cells. *n* = 50 cells. Quantitative graph is represented as mean of 50 cells (black dots) from three independent wells. p‐values were calculated using two‐tailed *T* test statistical analysis. Scale bar = 200 μm. *p* values were calculated using two‐tailed *T* test statistical analysis. **H** FACS-based cell cycle analysis and quantification of percentage of cells in G2 phase for lentivirally transduced A431 cells (shRNA-control, shRNA USP28#1 or ∆NP63#1) upon exposure to either DMF or 5 µM CPPD for 48 h. Representative cell cycle profile in Fig. S4F. Quantitative graphic is represented as mean of three independent biological replicates (black dots). *p* values were calculated using two‐tailed *T* test statistical analysis. **I** CPPD pulse chase experiment, followed by immunoblotting against endogenous phospho-TP53 (Serine 15) and ɣ-H2AX in sh-control (NTC), sh-USP28#1 or sh∆NP63#1 A431 cells. A431 cells were treated with either DMF (−) or 5 µM CPPD for 1 h and collected at indicated time points after CPPD exposure. Alternatively, sh-NTC cells were cultured in the presence of 15 µM AZ1 at time of 5 µM CPPD addition, and continuously after CPPD washout. ACTIN served as loading control (*n* = 3). Representative immunoblots of *n* = 3. Relative amount of protein abundance and standard deviation (SD) are presented from *n* = 3 experiments. Relative protein intensities were quantified respect to sample “sh-NTC -”(DMF treated sh-NTC A431 cells) upon ACTIN normalization. Bar graphs show the mean quantification of phospho-TP53 and ɣ-H2AX in three biological replicates (black dots) of A431 cells at 9 h post CPPD exposure. *p* values were calculated using two‐tailed *T* test statistical analysis. See also Fig. S4.

To determine whether elevated levels of USP28 confer resistance to chemotherapy, we ectopically expressed USP28 or ∆Np63 in a chemosensitive cell line, BEAS-2B [34], (Fig. 4A) and exposed cells to increasing concentrations of CPPD (Figs. 4B and S4A). Control cells were sensitive to CPPD and prone to DNA damage, indicated by elevated levels of ɣ-H2AX. Overexpression of either USP28 or ∆Np63 increased cell survival and decreased ɣ-H2AX levels (Figs. 4B–D and S4A). Similar results were obtained in the USP28 mutant cell line SK-MES1 (Fig. S4B). Next, we targeted USP28 and its substrate ∆Np63, respectively, by shRNA in A431 cells (Fig. S4C) and assessed cell viability after treatment with CPPD (Fig. 4E). Knock-down of USP28 or ∆Np63 significantly lowered the GI_50_ towards CPPD from 5.8 µM to 3 µM or 2.7 µM, respectively. Comparable values were found for A431 cells exposed to AZ1 (Fig. 4E). Of note, expression of sh-USP28 and sh-∆Np63 increased levels of ɣ-H2AX even without treatment (Fig. 4F and S4D). Similar observations were obtained for the USP28 SQ/TQ-mutants, supporting a role of USP28 in the ATM/ATR-dependent stress response (Fig. 2C, D) [25, 26].

Immunostaining for TP53BP1 provided further evidence for increased replicational stress after exposure to CPPD. Both, USP28-inhibited and USP28 or ∆Np63 knock-down A431 cells display high numbers of TP53BP1 foci in untreated conditions, which are further increased in response to CPPD exposure (Fig. S4E). Consistent with these observations, CPPD induced a potent G2-phase arrest in knockdown or inhibitor-treated cells and a weaker arrest in control cells (Fig. 4H and S4F). To examine the time-dependent DNA damage response of these cells, we exposed them to CPPD for 1 h prior release, washed, and probed lysates harvested at different time points afterwards for TP53 phosphorylation and ɣ-H2AX by immunoblotting (Fig. 4I). In control cells, both TP53 and H2AX were phosphorylated rapidly upon CPPD treatment and the signal diminished within 9 h post pulse. In contrast, USP28 knock-down and AZ1-inhibited A431 cells maintained high levels of phosphorylated TP53 and ɣ-H2AX up to 9 h post CDDP. Similar results were obtained upon knock down of ∆Np63 (Fig. 4I).

These data demonstrate that USP28, potentially via ∆Np63, facilitates CPPD resistance and is involved in DNA damage repair upon chemotherapy treatment.

### Disrupting the USP28-∆Np63 axis affects Fanconi anemia DDR signature genes

**Fig. 5:**
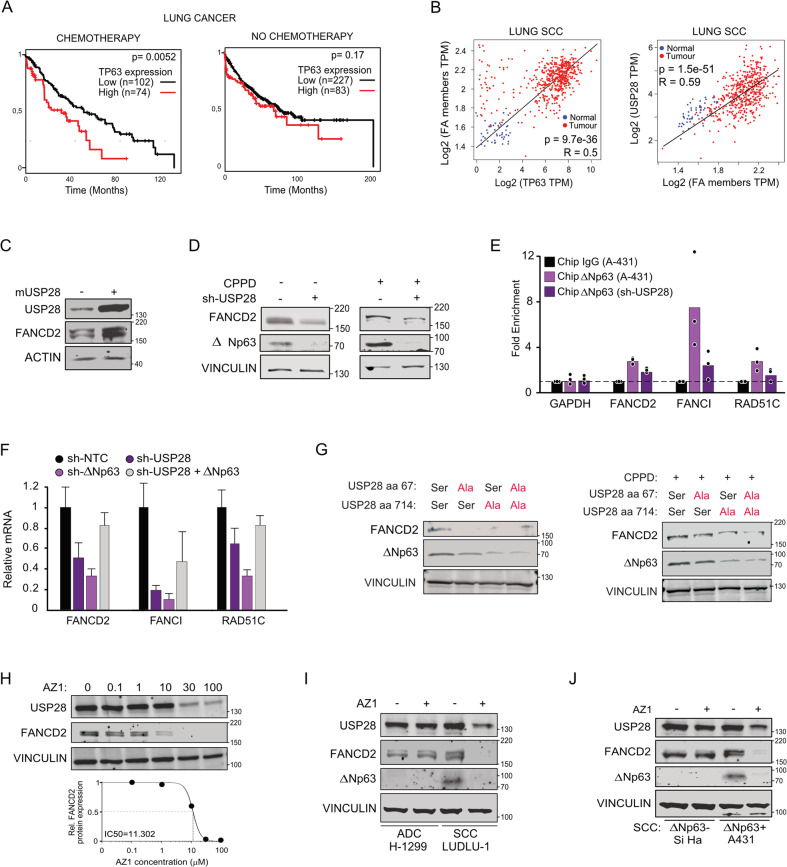
Deregulation of ∆Np63 impairs the Fanconi anemia pathway in SCC. **A** Publicly available patient survival data of NSCLC upon stratification towards relative expression of ∆NP63 and application of Chemotherapy. Left panel= NSCLC patients treated with chemotherapy (*n* = 176); Right panel= NSCLC patients non-treated with chemotherapy (*n* = 310). Generated with the online tool www.kmplot.com. **B** Correlation of gene expression between TP63 or USP28 and FA signature in human lung SCC (red dots) and normal tissue (blue dots). The diagonal line reflects a regression build on a linear model. R: Spearman’s correlation coefficient. *n* = 536. Generated with the open source tool www.gepia2.cancer-pku.cn. **C** Immunoblot of USP28 and FANCD2 in A431 cells virally transduced with doxycycline inducible overexpression of murine Usp28. Cells were exposed to 1 µg/ml Doxycycline for 96 h prior to analysis. ACTIN serves as loading control. Representative immunoblot of *n* = 3. **D** Immunoblot of endogenous USP28, FANCD2 and ∆NP63 in A431 cells harbouring a conditional shRNA targeting USP28. Cells were exposed to DMF (CPPD-) or 5 µM CPPD for 6 h. VINCULIN serves as loading control. Representative immunoblot of *n* = 3. **E** Chromatin immuno-precipitation of either IgG (black) or endogenous ∆Np63 in sh-NTC and sh-USP28#1 A431 cells, followed by RT-PCR of *GAPDH*, *FANCD2*, *FANCI* and *RAD51C* promotor regions using primers flanking the putative ∆Np63 promotor binding sites. Normalized to IgG. Quantitative graphic is represented as mean of three independent biological replicates (*n* = 3; red dots). **F** Quantitative RT-PCR of *FANCD2, FANCI* and *RAD51C* relative mRNA expression in A431 cells stably transduced with either sh-NTC, sh-USP28#1, sh-∆Np63#1 or sh-USP28#1 transiently transfected with ∆Np63 (gray) to rescue loss of ∆Np63 protein abundance upon knock down of USP28. Values are presented as normalized to ACTB. Quantitative graphic is represented as mean and SD of three independent biological replicates (*n* = 3). **G** Immunoblot of endogenous FANCD2 and ∆NP63 in control, S67A, S714A and compound S67A + S714A mutant A431 cells treated with either solvent control (DMF) or 5 µM CPPD for 6 h. VINCULIN serves as loading control. Representative immunoblots of *n* = 3. **H** Immunoblot of endogenous USP28 and FANCD2 in A431 cells treated for 24 h with either DMSO or indicated concentrations of AZ1. VINCULIN served as loading control. FANCD2 half‐maximal inhibitory protein abundance (IC_50_) was calculated. Representative immunoblots of *n* = 3. **I** Immunoblot of USP28, FANCD2 and ∆NP63 in control or AZ1 (15 µM) 24 h treated lung cells H1299 (ADC) and LUDLU‐1 (SCC). VINCULIN served as loading control. Representative immunoblots of *n* = 3. **J** Immunoblot of USP28, FANCD2 and ∆Np63 in cervix SiHa (∆Np63-) and vulva A431 (∆Np63+) cells treated with DMSO or AZ1 (15 µM) for 24 h. VINCULIN served as loading control. Representative immunoblots of *n* = 3. See also Figs. S5, S6.

Lung SCCs express elevated levels of USP28, ∆Np63 and several members of FA pathway genes (Fig. S5A), in agreement with a previous study which showed that ∆Np63 activates the Fanconi Anemia pathway [23]. We wondered if targeting the USP28-∆Np63 axis re-establishes chemotherapy sensitivity (Fig. 5A and [35]). Of note, high levels of ∆Np63 and USP28 in lung SCCs show a direct correlation with an increased expression of FA genes (Fig. 5B). In vitro, FANCD2 abundance depends on ∆Np63 as indicated by knock-down of ∆Np63 by two independent shRNA in A431 cells (Fig. S5B). CPPD pulse- chase experiments in A431 cells demonstrated that both ∆Np63 and FANCD2 levels increased after CPPD treatment, reaching its peak after 6 h (Fig. S5C). Importantly, most of the available FANCD2 was monoubiquitylated, hence active, as indicated by the upper band. Cells depleted of ∆Np63 failed to increase FANCD2 levels and to efficiently activate FANCD2 upon CPPD treatment (Fig. S5C). Loss of ∆Np63 reduced expression of FA genes, as seen by RNA sequencing and mass spectrometry analysis (Figure S5D–G). Conditional expression of murine USP28 in A431 cells enhanced protein abundance of FANCD2 (Fig. 5C). Conversely, shRNA-mediated knock-down of USP28 decreased FANCD2 protein abundance (Fig. 5D).

Chromatin immunoprecipitation of endogenous ∆Np63 in control and USP28 knock-down A431 cells showed that ∆Np63 was located at the promoters of FANCD2, FANCI and RAD51C and that loss of USP28 significantly reduced ∆Np63 binding to these promoters (Fig. 5E). This resulted in a significant reduction in the mRNA expression of FANCD2, FANCI and RAD51C (Fig. 5F). Overexpression of exogenous ∆Np63 partially restored the expression of FANCD2, FANCI and RAD51C in USP28 knock down cells, confirming that USP28 affects the FA pathway via ∆Np63, (Figs. 5F and S5H). Consistently, USP28 knock-down cells failed to upregulate FANCD2 and ∆Np63 upon CPPD treatment (Fig. 5D). Similar effects were observed in the ATR SQ/TQ motif mutant A431 cells (Fig. 5G).

Furthermore, exposure of A431 cells to increasing concentrations of AZ1 resulted in reduction of FANCD2 levels in a concentration-dependent manner (Fig. 5H). This effect was also observed in multiple SCC cell lines (Fig. S6A). To investigate if this effect is SCC specific, we compared the expression of FANCD2 upon treatment with AZ1 in a lung adenocarcinoma cell line, NCI-H1299, versus an SCC cell line, LUDLU-1 (Fig. 5I). Furthermore, to identify if the effect of USP28 inhibition is via ∆Np63, we compared the two SCC lines Si Ha (∆Np63^negative^) and A431 (∆Np63^positive^) (Fig. 5J). While FANCD2 was detectable in all tested cell lines, only ∆Np63 expressing cells lost FANCD2 expression upon exposure to AZ1, along with ∆Np63 itself (Fig. 5I, J).

Next, we performed a CPPD pulse chase experiment to investigate if USP28 determines the activity of the FA pathway upon CPPD exposure. Similarly, to our observation in ∆Np63 depleted A431 cells (Fig. S5C), sh-USP28 or AZ1 treated cells failed to increase FANCD2 protein abundance upon 6 h post-CPPD (Fig. S6B), implying a connection between USP28-∆Np63 and the FA pathway in SCC cells.

### Pharmacologic inhibition of USP28 sensitizes SCC cells to chemotherapy

By treating several human cancer cell lines with CPPD, we could observe that SCC, and in particular ∆Np63 expressing cells, were less sensitive to Cisplatin compared to ADC cell lines (Figs. S7A and S7B).

**Fig. 6:**
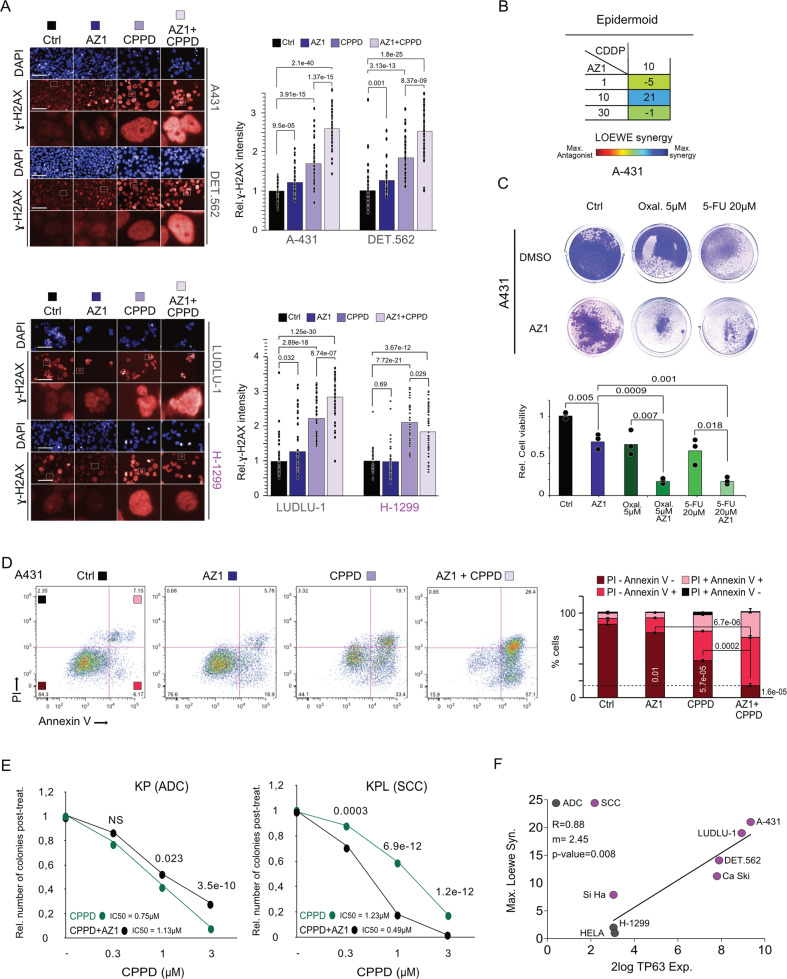
Pharmacologic inhibition of USP28 re-sensitizes SCC cells to chemotherapy. **A** Immunofluorescence staining of ɣ-H2AX in A431, Detroit 562, H-1299 and LUDLU-1 cells treated with DMSO + DMF, AZ1 + DMF (Ctrl), CPPD + DMSO or AZ1 + CPPD. A431, DETROIT 562 (DET.562) and H-1299 cells were treated with 15 µM AZ1, 5 µM CPPD or 15 µM AZ1 + 5 µM CPPD for 48 h. LUDLU-1 cells were treated with 1 µM AZ1, 5 µM CPPD or 1 µM AZ1 + 5 µM CPPD. DAPI served as nuclear marker. Relative quantification of the ɣ-H2AX staining intensity was measured for the different treatment exposures. Quantitative graphic is represented as median of 50 cells (black dots) from three independent wells. *p* values were calculated using two‐tailed *T* test statistical analysis. *n* = 50 cells. Scale bar = 200 μm. Red = SCC cell line; Blue = ADC cell line. **B** LOEWE synergism score of CPPD and AZ1 in A431 cells. Cells were exposed to 10 µM CPPD and indicated concentrations of AZ1 (1, 10, 30 µM) for 48 h. DAPI was used to assess total cell numbers and quantify LOEWE synergism using Combenefit software. Red = SCC cell line; Blue = ADC cell line. **C** Crystal violet cell viability assay. Viability assay was performed in A431 cells upon treatment with DMSO (Ctrl), 5 µM Oxaliplatin, 20 µM 5-FU, 15 µM AZ1 + 5 µM Oxaliplatin, 15 µM AZ1 + 20µM5-FU for 48 h. Representative image of *n* = 3. Quantitative graphic is represented as mean of three independent biological replicates (red dots). *p* values were calculated using two‐tailed *T* test statistical analysis. **D** Propidium Iodide (PI)/Annexin V FACS analysis to assess cell survival and apoptosis of A431 cells, treated with either DMSO/DMF (Control), 15 µM AZ1, 5 µM CPPD or the combination thereof for 48 h. Bar graph represents the relative amount of PI-/Annexin V- cells, PI-/Annexin V + cells, PI + /Annexin V- cells and PI + /Annexin V + cells. Quantitative graphic is represented as mean and standard deviation (SD) of three independent biological replicates. Representative plot of *n* = 3. *p* values were calculated using two-tailed *T* test statistical analysis. **E** Relative number of crystal violet stained colonies upon treatment with either DMSO or 15 µM AZ1 for 48 h and co-exposure to CPPD or DMF for 24 h at indicated concentrations in the murine KP (ADC) and KPL (SCC) cell lines. The experiment was performed as described in Fig. S9B. Quantitative graphic is represented as mean of *n* = 11. *p* values were calculated using two‐tailed *T* test statistical analysis. **F** Spearman correlation with TP63 mRNA expression of the indicated cell lines (Fig. S7A) and the maximum LOEWE synergism (Fig. 6B and S7D). TP63 mRNA expression were obtained from www.r2.amc.nl. Loewe synergy was calculated using Combenefit software. The diagonal line reflects a regression build on a linear model. R: Spearman’s correlation coefficient, m: slope of the linear regression mode. See also Figs. S7, S8, and S9.

If ∆Np63 mediates CPPD resistance, and USP28 regulates ∆Np63 protein abundance, treatment with AZ1 should synergize with CPPD. To test this hypothesis, we exposed human SCC and, where applicable, same tissue ADC cells to various concentrations of AZ1 and CPPD (Figs. 6A, B and S7C–E). Cells were treated for 48 h followed by nuclear staining with DAPI and immunofluorescence staining against the DNA damage marker ɣ-H2AX (Figs. 6A and S7C). While ∆Np63-negative cell lines showed no additive effect on cell viability upon co-treatment with CPPD and AZ1, ∆Np63 expressing SCC cell lines showed synergistic effects when combining both compounds (Figs. 6B and S7D). Moreover, treatment with AZ1 sensitized SCC cells not only to CPPD, but also to Oxaliplatin and 5-FU (Fig. 6C).

To test if ∆Np63 is mediating chemoresistance, we next treated an ADC cell line that expresses only low levels of ∆Np63, A549, as well the ∆Np63 negative SCC line Si-Ha with CPPD, AZ1 or the combination of both compounds (Fig. S7C). After treatment with both compounds, Si-Ha, despite being of SCC origin, responded to the combinatorial treatment similar to ADC lines (Fig. S7C, D). In contrast, A549 cells increased ɣ-H2AX expression and showed less cell survival under combinatorial treatment, thereby responding similar to SCC cell lines (Fig. S7C, E). This observation demonstrates that expression of ∆Np63 strongly determines sensitivity towards USP28 inhibition.

To investigate if co-exposure of the ∆Np63 positive cell line A431 to AZ1 and CPPD induces cell death, we treated this cell line to either control solvent alone (DMF/DMSO), AZ1, CPPD or a combination and analysed cell viability after 48 h by PI incorporation and anti-Annexin V staining (Fig. 6D). AZ1 alone resulted in a mild increase of early apoptotic cells (Fig. 6D). Exposure to CPPD resulted in a marked increase in early and late apoptotic cells (Fig. 6D). Co-exposure of A431 cells to AZ1 and CPPD shifted the majority of cells into an apoptotic state (Fig. 6D). This selective sensitivity was seen in various ∆Np63 positive cells, where co-treatment of AZ1 and CPPD resulted in increased levels of apoptosis (Fig. S8A). Consistently, knock down of USP28 or ∆Np63 in A431 cells resulted in reduced vitality pre and post CPPD exposure, indicated by cell counts and number of cleaved caspase 3 positive cells (Fig. S8B–D).

To further validate the AZ1-mediated sensitivity we examined our previously established murine primary lung cancer cell lines, the ADC cell line KP (∆Np63^negative^, *Kras*^*G12D*^*, Trp53*^*∆*^) and the SCC cell line KPL (∆Np63^positive^, *Kras*^*G12D*^*, Trp53*^*∆*^, *Lkb1*^*∆*^) (Fig. S9A) [24, 36]. In agreement with our previous finding, KPL cells maintained a higher colony formation capacity than KP cells upon CPPD treatment (Fig. S9B–D), but co-exposure with AZ1 sensitized KPL cells and led to a significant decrease in colony formation capacity that was not observed for KP (Fig. 6E). Notably, AZ1 caused already at 1 µM CPPD low cell survival for KPL cells. At 3 µM CPPD the line KPL almost succumbed to the treatment (Figs. 6E and S9C).

Hence, inhibition of USP28 synergizes with CPPD predominantly in cells expressing ∆Np63, while in ADC or SCC cells lacking ∆Np63 expression, no cooperation between CPPD and USP28 inhibition could be observed (Fig. 6E, F).

### Inhibition of USP28 activity deregulates FA-DDR signaling and sensitizes tumors to CPPD treatment

To assess the role of USP28 activity on the FA pathway in vivo, we used a CRISPR/Cas9 expressing mouse strain, in combination with AAV virions, for tumor induction and depletion of USP28 in the lung (Fig. S10A, B) [24, 36, 37]. Immuno-histochemical analysis of lung sections of KPL mice showed that USP28 was readily detectable, and the DDR markers TP53BP1, ɣ-H2AX and the FA effector FANCD2 were expressed (Fig. S10C). In tumors depleted of Usp28 via two sgRNA (KPLU), the DNA damage sensors (TP53BP1, ɣ-H2AX) were strongly upregulated, while FANCD2 was not detectable (Fig. S10C). Western blot analysis of primary tumor material comparing KPL and KPLU showed the expected depletion of USP28 and loss of ∆Np63 (Fig. S10D) [24]. In KPLU tumors, the overall protein abundance of the FA proteins FANCD2 and FANCI were significantly reduced compared to USP28-proficient tumor samples (Fig. S10D), demonstrating that the USP28-∆Np63 axis maintains FA expression in vivo.

**Fig. 7:**
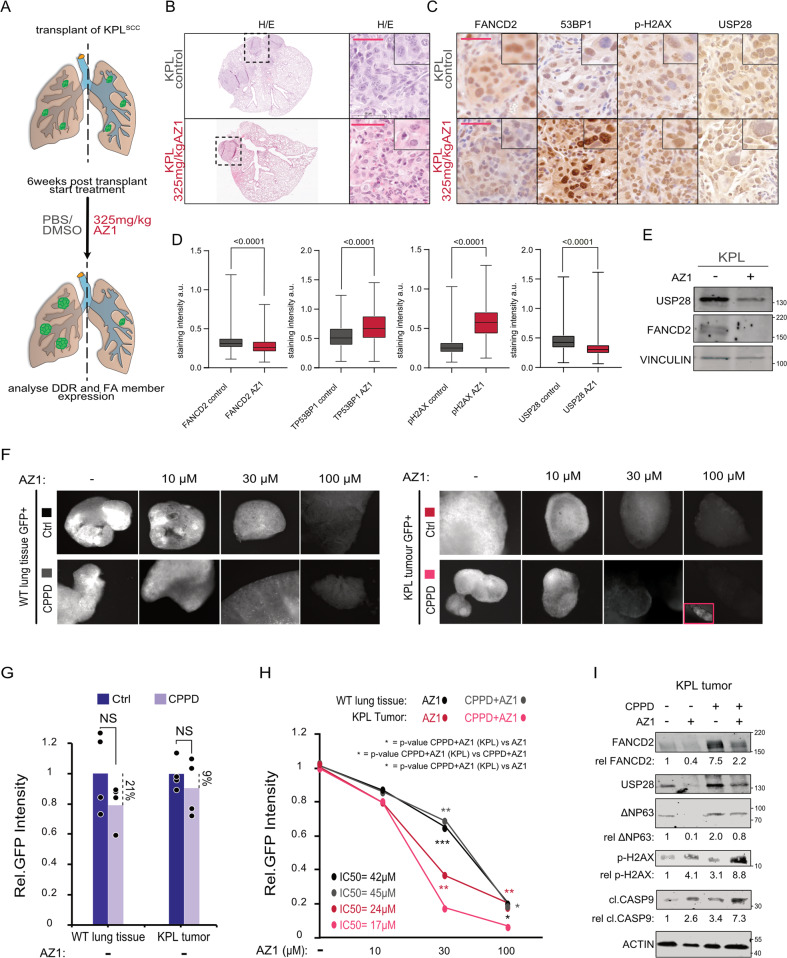
Inhibition of USP28 activity deregulates FA-DDR signaling in vivo and sensitizes tumors to CPPD treatment in ex vivo organotypic lung SCC tumor slice cultures by de-activating FA. **A** Schematic diagram of isogenic transplant experiment of of *KRas*^*G12D*^*:Trp53*^∆^:*Lkb1*^∆^ (*KPL*) cells, followed by treatment with either vehicle (PBS/DMSO/Tween) or AZ1 (375 mg/kg), for a total of 18 days as previously reported [24]. **B** Representative H&E stained sections from KPL transplant tumors upon treatment with either vehicle (PBS/DMSO/Tween) or AZ1 (375 mg/kg), for a total of 18 days. Inlay shows higher magnification. *n* = 3. Scale bar = 50 μm. **C** Immunohistochemistry of FANCD2, TP53BP1, ɣ-H2AX and USP28 in KPL transplant tumors post treatment with either vehicle (PBS/DMSO/Tween) or AZ1 (375 mg/kg), for a total of 18 days. *n* = 3. Scale bar = 50 μm. **D** Quantification of relative immunohistochemical staining intensity of FANCD2, ɣ-H2AX, TP53BP1 and USP28 in vehicle or AZ1 treated mice. Statistical analysis was performed using unpaired *t* test. *p* < 0.0001. Images were quantified using QuPath (version0.2.3). Boxplots were generated using Graphpad Prism8. a.u.= Arbitrary units. In box plots, the centre line reflects the median and the upper and lower box limits indicate the first and third quartiles. Whiskers extend 1.5× the IQR.n^FANCD2^ = 6200 control/3616 AZ1; *n*^ɣ-H2AX^ = 17075control/9140 AZ1; *n*^TP53BP1^ = 18831 control/8586 AZ1; *n*^USP28^ = 13287control/8329 AZ1. **E** Immunoblot of endogenous USP28 and FANCD2 from KPL tumors post treatment with either vehicle (PBS/DMSO/Tween) or AZ1 (375 mg/kg), for a total of 18 days. VINCULIN served as loading control. Representative immunoblots of three independent biological replicates (*n* = 3). **F** Immunofluorescence of either WT GFP^+^ lung cells or KPL (*KRas*^*G12D*^*:Trp53*^∆^:*Lkb1*^∆^) GFP^+^ lung tumor cells, after orthotopic re-transplantation in wild type C57BL6/J mice, within the organotypic slice culture after 72 h of indicated AZ1 concentrations (0, 10, 30, 100 μM), followed by 24 h of co-treatment with either solvent control (DMF) or 5 µM CPPD. Representative images of four independent biological replicates (*n* = 4). **G** Quantification of relative GFP^+^ signal intensity of either WT GFP^+^ lung cells or KPL (*KRas*^*G12D*^*:Trp53*^∆^:*Lkb1*^∆^) GFP^+^ lung tumor cells, after orthotopic re-transplantation in wild type C57BL6/J mice, within the organotypic slice culture after 24 h of co-treatment with 5 µM CPPD. Representative images of four independent biological replicates (*n* = 4). Quantitative graph is represented as mean of *n* = 4 (black dots). *p* values were calculated using two‐tailed *T* test statistical analysis. **H** Quantification of relative GFP^+^ signal intensity of either WT GFP^+^ lung cells or KPL (*KRas*^*G12D*^*:Trp53*^∆^:*Lkb1*^∆^) GFP^+^ lung tumor cells, after orthotopic re-transplantation in wild type C57BL6/J mice, within the organotypic slice culture after 72 h of indicated AZ1 concentrations (0, 10, 30, 100 μM), followed by 24 h of co-treatment with either solvent control (DMF) or 5 µM CPPD. Representative images of four independent biological replicates (*n* = 4). Quantitative plot is represented as mean of *n* = 4. *p* values were calculated using two‐tailed *T* test statistical analysis. *p < 0.05; **p < 0.01; ***p < 0.001. Exact *p* values in Fig. S11B. **I** Immunoblot of FANCD2, ɣ-H2AX, ∆NP63, USP28, cleaved Caspase 9 and ACTIN in KPL (*KRas*^*G12D*^*:Trp53*^∆^:*Lkb1*^∆^) GFP^+^ lung tumor cells within the organotypic slice culture after 72 h of 30 µM AZ1 concentrations and 24 h of co-treatment with 5 µM CPPD. ACTIN serves as loading control. Representative immunoblots of four independent biological replicates (*n* = 4). Relative protein intensities were quantified respect to control sample (CPPD− and AZ1−) upon ACTIN normalization. See also Figs. S10 and S11.

As systemic inhibition of USP28 is well tolerated in mice [24], we wondered if deregulated DDR via inhibition of the FA pathway could be observed in AZ1-treated animals (Fig. 7A, B). Immuno-histochemical analysis of tumor-bearing lungs from murine SCC transplant animals revealed that in control treated animals USP28 and its substrate ∆Np63 were detectable, along with the ∆Np63 target FANCD2 (Figs. 7C and S10E). DNA damage markers, such as TP53BP1 and ɣ-H2AX, were only weakly expressed (Fig. 7C). Exposure of animals to AZ1 reduced levels of USP28 and ∆Np63, as previously described [24] (Figs. 7C and S10E). Compared to control animals, FANCD2 was only weakly expressed and the DDR markers TP53BP1 and ɣ-H2AX upregulated (Fig. 7C, D). Similarly, tumour tissue explants revealed a significant reduction in USP28 and FANCD2 upon treatment with AZ1 (Fig. 7E).

We next tested different treatment regimes in A431 cells for using AZ1 and CPPD, comprising single compound treatment, AZ-1/CPPD co-treatment, AZ-1 pre-treatment followed by CPPD and AZ-1 pre-treatment, followed by AZ-1/CPPD co-treatment (Fig. S10F–H). AZ-1 showed the weakest DDR staining intensity, followed by cells exposed to CPPD alone. Pre-treatment with AZ-1 followed by co-exposure to the small molecule inhibitor and the chemotherapeutic agent resulted in a strong upregulation of TP53BP1, including an even distribution within the nucleus, and led to an enhanced cell death in A431 cells (Fig. S10F–H).

To investigate a potential therapeutic synergism between AZ-1 and CPPD in a multicellular system, we decided to employ the ex vivo organotypic lung slice culture (Fig. S11A). Here, isogenic murine SCC cells (KPL) were orthotopically re-transplanted in immune-competent C57BL6/J mice where they engrafted and formed a tumor (Fig. S11A). 8 weeks’ post-transplant, mice were sacrificed and the tumor-bearing lungs explanted, following live tissue sectioning with a vibratome (Fig. S11A). As a control, we used a lung slice culture from a wild type C57BL6/J-*Rosa26*^*Sor-CAGG-Cas9-IRES-eGFP*^ mouse (Fig. S11A). Slices containing tumor (GFP^+^) or wild type tissue (GFP^+^) were cultured and exposed to small molecule inhibitor AZ1 (0–100 µM) and CPPD (5 µM) (Fig. S10F–H). Notably, SCC tumors presented a strong resistance to CPPD single treatment when compared to wildtype lung tissue (Fig. 7F, G). Exposure to AZ-1 alone significantly affected GFP + KPL cells, and co-treatment of the organotypic slice culture with AZ-1 and CPPD significantly reduced the amount of detectable and viable tumor cells at around ~30 µM AZ1/ 5 µM CPPD (Figs. 7F–H and S11B). In contrast, wild type lung slice cultures exposed to the same treatment regime tolerated these dosages significantly better and showed only minor responses (Figs. 7F–H and S11B). Immunoblotting of tissue samples from tumor-bearing organotypic slice cultures post treatment revealed that AZ-1 single treatment efficiently reduced FANCD2 protein abundance, and upon co-treatment with CPPD, SCC tumors lost FANCD2 and significantly upregulated ɣ-H2AX along with pro-apoptotic signaling, as seen by Caspase 9 cleavage, when compared to CPPD single treatment (Fig. 7I).

These data show that inhibition of USP28 specifically affects tumor cell growth and the DDR response. Priming of SCC cells via AZ-1 potentiates the therapeutic efficacy of AZ-1/CPPD co-treatment in vitro and ex vivo.

## Discussion

Several therapeutic strategies aim at inflicting DNA damage to overwhelm the DNA damage repair machinery in tumor cells, as these cells, in contrast to non-transformed cells, frequently harbor mutations in check point genes and fail to halt the cell cycle to initiate the repair of damaged DNA [38–41]. Deubiquitylating enzymes are involved in the DNA damage response pathway, including USP28, which is recruited to sites of ionizing radiation induced DNA damage, where it interacts with TP53BP1 and ATM [26, 42, 43]. Interaction with ATM contributes to the binding of USP28 to TP53BP1, the overall role of USP28 in DNA damage signaling, however, was unclear [25].

In this study we showed that USP28 is recruited to sites of cisplatin-induced DNA damage, resulting in an interaction with ATR and phosphorylation of USP28. The subsequent phosphorylation of USP28 resulted in an increased USP28 enzymatic activity and stabilization of USP28 substrates, such as MYC and ∆Np63. Inhibition of ATR resulted in the degradation of USP28, as the protein stability of DUBs is linked to their activity [44, 45]. In SCC, ∆Np63 is an essential factor regulating chemoresistance by controlling the expression of DNA repair genes [17, 46]. Both knock-down or pharmacologic inhibition of USP28 reduced ∆Np63 protein levels and its downstream targets, including the DNA repair proteins FANCD2, FANCI or RAD51C. As a consequence, in ∆Np63 expressing SCC, inhibition of USP28 synergized with cisplatin, resulting in enhanced DNA damage and reduced overall SCC survival in a dose-dependent fashion. Notably, inhibition of USP28 in human adenocarcinoma cell lines, and in the SCC line SiHa, which does not express ∆Np63, led to a reduction of ɣ-H2AX, and co-treatment with cisplatin had no additive nor synergistic effect. Similar observations were made in other tumor entities, where loss of USP28 induced treatment resistance [47].

Targeting USP28, by shRNA depletion or pharmacological inhibition, resulted in a reduced tumor burden in an in vivo lung tumour model [24]. Loss or impairment of USP28 in ex vivo and in vivo models of NSCLC resulted in a significant increase in DNA damage marker abundance and reduction of the FA pathway member FANCD2. The combination of USP28 inhibition and cisplatin further induced DNA damage, leading to tumor shrinkage, while wild type tissue tolerated the treatment.

USP28 has been reported to regulate TP53 abundance in a USP28-TP53BP1 cell cycle-dependent fashion [48–50]. Since mutational load of TP53 and cell cycle state directly affect the response to cisplatin treatment in cancer [51], the observed AZ-1 mediated sensitivity may function via TP53. However, the majority of patients diagnosed with (lung) squamous cell carcinoma harbour inactivating or LOF mutations within TP53 [3, 24]. These reports argue that the observed effects upon interference with USP28 on cell proliferation and cisplatin response in SCC are independent of TP53.

USP28 behaves as a non-oncogene addiction gene, since wild type cells tolerate its inactivation, while tumor cells, SCC in particular, depend on its expression [52, 53]. Based on these findings, USP28 presents a promising therapeutic target, in combination with DNA-damaging agents such as CPPD, in SCC; while in ADC, due to the ∆Np63-independent expression of FA proteins by the E2F family [54], targeting of USP28 could have adverse effects and even support the establishment of therapy resistance. Overall, our results show that targeting the USP28-∆Np63 axis in SCC tones down the FA-DDR pathway, thereby sensitizing SCC to Cisplatin treatment.

## Material and methods

### Tissue culture and reagents

A431, Beas-2B, SiHa, Ca SKI, DETROIT 562, HEK-293T, NCI-H1299. cell lines were obtained from ATCC or ECACC. The human lung cancer cell line LUDLU-1^adh^ was described previously [24]. A431, Beas-2B, SiHa, Ca SKI, DETROIT 562 and HEK-293T cells were cultured in DMEM (Gibco) supplemented with 10% fetal bovine serum (FCS)/ 1% Pen-Strep at 37 °C and 5% CO_2_ in a humidified environment. LUDLU-1^adh^, NCI-H1299, CALU 1, SK-MES1, and H23 cells were cultured in RPMI 1640 (Gibco) supplemented with 10% FCS/ 1% GlutaMAX/ 1% Pen Strep. Cell lines were authenticated by STR profiling. Cells were routinely tested for mycoplasma via PCR.

Except when a different concentration was expressly indicated, the reagents were dissolved in Dimethyl sulfoxide (DMSO) or Dimethylformamide (DMF) and added to the cells at the following concentrations: Cisplatin (CPPD; 5 μM; dissolved in DMF), doxycycline (DOX; 1 μg/ml), Tandem ubiquitin binding entity (TUBE; 100 μg/ml), KU55933 (15 µM; dissolved in DMSO) and VE 821 (2.5 µM; dissolved in DMSO).

### DNA transfection and infection

DNA transfection was performed adding a mix of 2.5 μg plasmid DNA, 200 μl serum free medium and 5 μl PEI to the cells seeded in a 6-well plate (60% confluence), after 6 h incubation at 37 °C the medium was changed to full supplemented medium and finally, cells were collected after 48 h for experimental purposes. For viral infection, AAVs or Lentiviruses (MOI = 10) were added to the medium in the presence of polybrene (5 μg/ml) and incubating at 37 °C for 4 days. The selection of infected cells was performed with 2,5 μg/ml Puromycin for 72 h, 250 µg/ml Neomycin for 2 weeks or FACS-sorting RFP/GFP positive cells (FACS Canto II BD).

### Primary murine lung cancer cell lines and colony formation assay

Primary lung cancer cell lines were obtained from 12 weeks old mice as previously described [24]. At endpoint of experiment, mice were sacrificed and lung tumors isolated. Tissue was digested in Collagenase I 100 U/ml in PBS for 30 min at 37 C and after stopping the reaction with FCS, the mixture was centrifuged and re-suspended in DMEM (Gibco) supplemented with 10% fetal bovine serum (FCS) and 1% Pen-Strep. Fibroblasts were counter-selected by selective trypsinisation and homogeneous cell clusters were clonally expanded. All clones have been characterized and classified according to markers as adenocarcinoma (KP cell lines) or squamous cell carcinoma (KPL cell line).

For colony formation assay, murine cells were treated at indicated concentrations of CPPD/AZ1 (Figs. 5G and S5H) for 3 days. After exposure, 400 cells were re-seeded in a new 10 cm plate and maintained in DMEM supplemented with 10% fetal bovine serum (FCS) and 1% Pen-Strep for 14 days. Number of healthy KP/KPL colonies was quantified manually upon staining with 0.5% Crystal violet.

### RT-PCR and CHIP-QPCR

RNA was isolated with Peq GOLD Trifast (Peqlab), as indicated in the manufacturer’s instructions. RNA was reverse transcribed into cDNA using random hexanucleotide primers and M-MLV enzyme (Promega). ChIP experiments were performed using 20 μg anti-ΔNp63 (Biolegend) as previously reported (Herold et al. 2019). Quantitative RT-PCR was performed with SYBR Green mix (ABgene) on the instrument “Step One Realtime Cycler”(ABgene) The RT-PCR program employed in this research is the following: 95 °C for 15 min., 40x [95 °C for 15 sec., 60 °C for 20 sec. and 72 °C for 15 sec.], 95 °C for 15 sec. and 60 °C for 60 sec. Relative expression was generally calculated with ΔΔCt relative quantification method. Melt curve was performed for all primers. Primers used for this publication are listed.

### Immunoblot, co-immunoprecipitation and chromatin fractionation

Cells have been lysed in RIPA lysis buffer (20 mM Tris-HCl pH 7.5, 150 mM NaCl, 1 mM Na^2^EDTA, 1 mM EGTA, 1% NP-40 and 1% sodium deoxycholate), containing proteinase inhibitor (1/100) via sonication with Branson Sonifier 250 (duty cycle at 20% and output control set on level 2; 10 sonication / 1 min cycles per sample). Protein concentration was quantified using Bradford assay as previously described [24]. A total of 50 μg protein was boiled in 5x Laemmli buffer (312.5 mM Tris-HCl pH 6.8, 500 mM DTT, 0.0001% Bromphenol blue, 10% SDS and 50% Glycerol) for 5 min and separated on 10% Tris-gels in Running buffer (1.25 M Tris base, 1.25 M glycine and 1% SDS). After separation, protein was transferred to Polyvinylidene difluoride membranes (Immobilon-FL) in Transfer Buffer (25 mM Tris base, 192 mM glycine and 20% methanol). Membrane was exposed to blocking buffer (0.1% casein, 0.2xPBS and 0.1% Tween20) for 45 min at room temperature (RT). Then, membranes were incubated with listed primary Abs (1/1000 dilution in a buffer composed by 0.1% casein, 0.2x PBS and 0.1% Tween20) for 6 h at room temperature (RT). Indicated secondary Abs (1/10000 dilution in a buffer composed by 0.1% casein, 0.2x PBS, 0.1% Tween20 and 0.01% SDS) were incubated for 1 h at RT. Membranes were recorded in Odyssey^®^ CLx Imaging System, and analysed using Image Studio software (Licor Sciences).

Immunoprecipitation was performed using 0.25 mg of Pierce™ Protein A/G Magnetic Beads (ThermoFisher), 1 μg of the listed specific Ab and 500 μg of protein lysate. For endogenous Co-Immunoprecipitations, beads were incubated with IgG (Sigma) as a control for specificity. Chromatin fractionation was performed following the manufacturer’s instructions and reagents of the Chromatin Extraction Kit (ab117152) (Abcam).

### Tandem-Ubiquitin-Binding-Entity assays

A431 cells were treated with 5 µM CPPD or solvent control for 6 h. Cells were harvested and lysed in RIPA + buffer (50 mM Tris-HCl pH 7.4, 1% NP-40, 0.5% Deoxychylate, 0.1% SDS, 150 mM NaCl, 2 mM EDTA, 5 mM MgCl2) supplemented with Protease-Inhibitor and 1 mM DTT. To ensure protection of ubiquitin-chains, chain-unspecific non-commercial GST-Tandem-Ubiquitin-Binding-Entities (TUBEs) must be added immediately at a concentration of 100 µg/mL to lysates. For pulldown experiments, cleared lysates were transferred to a new reaction-tube. Approximately 10% of the lysate was kept as “Input” sample, supplemented with 5x Laemmli-buffer and boiled for 5 min. The remaining lysates was used to enrich ubiquitylated proteins. GST-TUBE was captured by adding washed Glutathione-Sepharose 4B beads (GE Healthcare) while rotating o/n at 4 °C. After incubation, beads were washed 4 times with ice-cold PBS-T (0.1% Tween20) and subsequently boiled in 20 µL 1x Laemmli buffer for 10 min. Eluates were applied to SDS-PAGE and Western Blotting and detection of ubiquitylated-proteins was performed with the Odyssey CLx Imaging System.

### Ubiquitin suicide probe/warhead DUB activity assays

Cells grown in one well of a 6-well plate were harvested and resuspended in two pellet volumes of ice-cold HR-buffer (50 mM Tris-HCl pH 7.4, 5 mM MgCl2, 250 mM Sucrose, 0.1% NP-40), supplemented with Protease-Inhibitor. Lysis was performed by three freeze-thaw cycles (liquid nitrogen, 37 C waterbath), followed by subsequent centrifugation. To profile DUB activity, 25 µg of cell lysate were transferred to a new reaction tube and volume adjusted to 16 µL with HR-buffer. 3 µL of a 1:1:1 mixture of Ub-VME, Ub-VS, Ub-PA suicide-probes (in 50 mM NaOAc, 5% DMSO) were added to the lysate and to adjust the pH, double the volume 50 mM NaOH compared to probes was added. Samples were mixed briefly and incubated for 1 h at 37 °C shaking. After addition of 6 µL of 5x Laemmli-buffer, samples were boiled for 5 min and applied to SDS-PAGE, followed by Western Blotting. For active DUBs, the resulting 8 kDa size-shift was analysed using the Odyssey CLx Imaging System.

### Immunohistochemistry and immunofluorescence

For IF and IHC, primary antibodies were incubated ON at 4 °C, followed by subsequent incubation with the secondary antibody for 1 h at room temperature. After antibody exposure, slides were washed twice with PBS. Stained samples were mounted with Mowiol^®^40-88. IHC were recorded using Pannoramic DESK scanner and analyzed with Case Viewer software (3DHISTECH). For IF, tissue-samples/cells were counterstained with 5 μg/ml DAPI for 15 min after secondary antibody application. IF stained slides were recorded using a FSX100 microscopy system (Olympus). For antibodies, manufacturer’s manuals and instructions regarding concentration or buffer solutions were followed. TP53BP1 foci were analysed in 5 regions of interest (ROI, 10 cells per field) using ImageJ. For ɣ-H2AX, nuclear intensity was measured using ImageJ or the Operetta CLS High-Content Analysis System (Perkin Elmer). Number of cells or fields analysed were indicated

### Cell viability, Operetta system and IC_50_/GI_50_ calculation

For cell viability, cells were stained with 0.5% Crystal violet and analyzed using ImageJ software (staining intensity is between 0 and 255). Upon quantification of the staining intensity, values were normalized to control. Number of cells was quantified using Operetta High-Content Imaging System (PerkinElmer) (number of DAPI + cells) or Invitrogen Countess II FL (number of cells after trypsinization) upon indicated treatments. For the Operetta High-Content Imaging System, cells were seeded in 384-well plates at equal density and exposed to indicated treatments. Then, cells were fixed using 4% PFA for 10 min and then, permealized using 0.5% Triton x100 in PBS for 5 min. Before quantification cells were stained with DAPI. Number of cells was determined counting the number of nucleus with the Harmony Software (Perkin Elmer). Loewe synergy as calculated using the Combenefit software as previously described (Di Veroli GY et al. 2016). For the quantification, unhealthy cells with modified nuclear morphology were excluded. IC_50_ was calculated and visualized using the website: www.aatbio.com.

### sgRNA and shRNA design

sgRNAs were designed using the CRISPRtool (https://zlab.bio/guide-design-resources). shRNA sequences were designed using SPLASH-algorithm (http://splashrna.mskcc.org/) [55] or the RNAi Consortium/Broad Institute (www.broadinstitute.org/rnai-consortium/rnai-consortium-shrna-library).

### AAV and lentivirus production and purification

Viruses were synthetized in HEK293-T cells. For AAV production, cells were co-transfected with the plasmid of interest (10 μg), pHelper (15 μg) and pAAV-DJ (10 μg) using PEI (70 μg). AAV Virus isolation from transfected cells was performed as previously described [24]. For Lentivirus production, HEK293 cells (70% confluence) were transfected with the plasmid of interest (15 μg), pPAX (10 μg) and pPMD2 (10 μg) using PEI (70 μg). After 96 H, the medium containing lentivirus was filtered (0.45 µM) and stored at −80 °C.

### In vivo experiments and histology

All in vivo experiments were approved by the Regierung Unterfranken and the ethics committee under the license numbers 2532-2-362, 2532-2-367, 2532-2-374 and 2532-2-1003. The mouse strains used for this publication are listed. All animals are housed in standard cages in pathogen‐free facilities on a 12‐h light/dark cycle with ad libitum access to food and water. FELASA2014 guidelines were followed for animal maintenance.

Adult mice were anesthetized with Isoflurane and intratracheally intubated with 50 μl AAV virus (3 × 10^7^ PFU) as previoulsy decribed [24]. Viruses were quantified using Coomassie staining protocol [56]. Animals were sacrificed by cervical dislocation and lungs were fixed using 5% NBF. For IHC and H & E, slides were de-paraffinized and rehydrated following the protocol: 2 × 5 min. Xylene, 2 × 3 min. EtOH (100%), 2 × 3 min. EtOH (95%), 2 × 3 min. EtOH (70%), 3 min. EtOH (50%) and 3 min. H_2_O. For all staining variants, slides were mounted with 200 μl of Mowiol^®^ 40–88 covered up by a glass coverslip. IHC slides were recorded using Pannoramic DESK scanner or using FSX100 microscopy system (Olympus) and analysed using Case Viewer software (3DHISTECH) and ImageJ. IF samples were recorded using FSX100 microscopy system (Olympus).

### RNA-sequencing

RNA sequencing was performed with Illumina NextSeq 500 as described previously [57]. RNA was isolated using ReliaPrep™ RNA Cell Miniprep System Promega kit, following the manufacturer’s instruction manual. mRNA was purified with NEBNext^®^ Poly(A) mRNA Magnetic Isolation Module (NEB) and the library was generated using the NEBNext^®^ UltraTM RNA Library Prep Kit for Illumina, following the manufacturer’s instructions.

### Sample preparation for mass spectrometry

The sample preparation was performed as described previously [58]. Briefly, lysates were precipitated by methanol/chloroform and proteins resuspended in 8 M Urea/10 mM EPPS pH 8.2. Concentration of proteins was determined by Bradford assay and 100 µg of protein per samples was used for digestion. For digestion, the samples were diluted to 1 M Urea with 10 mM EPPS pH 8.2 and incubated overnight with 1:50 LysC (Wako Chemicals) and 1:100 Sequencing grade trypsin (Promega). Digests were acidified using TFA and tryptic peptideswere purified by tC18 SepPak (50 mg, Waters). A total of 125 µg peptides per sample were TMT labeled and the mixing was normalized after a single injection measurement by LC-MS/MS to equimolar ratios for each channel. 250 µg of pooled peptides were dried for offline High pH Reverse phase fractionation by HPLC.

### Offline high pH reverse phase fractionation

Peptides were fractionated using a Dionex Ultimate 3000 analytical HPLC. 250 µg of pooled and purified TMT-labeled samples were resuspended in 10 mM ammonium-bicarbonate (ABC), 5% ACN, and separated on a 250 mm long C18 column (X-Bridge, 4.6 mm ID, 3.5 µm particle size; Waters) using a multistep gradient from 100% Solvent A (5% ACN, 10 mM ABC in water) to 60% Solvent B (90% ACN, 10 mM ABC in water) over 70 min. Eluting peptides were collected every 45 s into a total of 96 fractions, which were cross-concatenated into 12 fractions and dried for further processing.

### LC-MS^3^ proteomics

All mass spectrometry data was acquired in centroid mode on an Orbitrap Fusion Lumos mass spectrometer hyphenated to an easy-nLC 1200 nano HPLC system using a nanoFlex ion source (ThermoFisher Scientific) applying a spray voltage of 2.6 kV with the transfer tube heated to 300 °C and a funnel RF of 30%. Internal mass calibration was enabled (lock mass 445.12003 m/z). Peptides were separated on a self-made, 32 cm long, 75 µm ID fused-silica column, packed in house with 1.9 µm C18 particles (ReproSil-Pur, Dr. Maisch) and heated to 50 °C using an integrated column oven (Sonation). HPLC solvents consisted of 0.1% Formic acid in water (Buffer A) and 0.1% Formic acid, 80% acetonitrile in water (Buffer B).

For total proteome analysis, a synchronous precursor selection (SPS) multi-notch MS3 method was used in order to minimize ratio compression as previously described (McAlister et al., 2014). Individual peptide fractions were eluted by a non-linear gradient from 4 to 40% B over 210 min followed by a step-wise increase to 95% B in 6 min which was held for another 9 min. Full scan MS spectra (350–1400 m/z) were acquired with a resolution of 120,000 at m/z 200, maximum injection time of 50 ms and AGC target value of 4 × 10^5^. The most intense precursors with a charge state between 2 and 6 per full scan were selected for fragmentation within 3 s cycle time and isolated with a quadrupole isolation window of 0.4 Th. MS2 scans were performed in the Ion trap (Turbo) using a maximum injection time of 50 ms, AGC target value of 1 × 10^4^ and fragmented using CID with a normalized collision energy (NCE) of 35%. SPS-MS3 scans for quantification were performed on the 10 most intense MS2 fragment ions with an isolation window of 1.2 Th (MS) and 2 m/z (MS2). Ions were fragmented using HCD with an NCE of 65% and analyzed in the Orbitrap with a resolution of 50,000 at m/z 200, scan range of 100–200 m/z, AGC target value of 1.5 × 10^5^ and a maximum injection time of 150 ms. Repeated sequencing of already acquired precursors was limited by setting a dynamic exclusion of 60 s and 7 ppm and advanced peak determination was deactivated.

### Quantification and statistical analysis

#### RNA-sequencing analysis

Fastq files were generated using Illuminas base calling software GenerateFASTQ v1.1.0.64 and overall sequencing quality was analyzed using the FastQC script. Reads were aligned to the human genome (hg19) using Tophat v2.1.1 [59] and Bowtie2 v2.3.2 [60] and samples were normalized to the number of mapped reads in the smallest sample. For differential gene expression analysis, reads per gene (Ensembl gene database) were counted with the “summarizeOverlaps” function from the R package “GenomicAlignments” using the “union”-mode and non- or weakly expressed genes were removed (mean read count over all samples <1). Differentially expressed genes were called using edgeR [61] and resulting *p* values were corrected for multiple testing by false discovery rate (FDR) calculations. GSEA analyses were done with signal2Noise metric and 1000 permutations. Reactome analysis were performed with PANTHER [62] using the “Statistical overrepresentation test” tool with default settings. Genes were considered significantly downregulated for reactome analysis when: Log2FC > 0.75 and FDR *p* < 0.05.

#### Proteomics analysis

Proteomics raw files were processed using proteome discoverer 2.2 (ThermoFisher). Spectra were recalibrated using the Homo sapiens SwissProt database (2018-11-21) and TMT as static modification at N-terminus and Lysines, together with Carbamidomethyl at cysteine residues. Spectra were searched against human database and common contaminants using Sequest HT with oxidation (M) as dynamic modification together with methionine-loss + acetylation and acetylation at the protein terminus. TMT6 (N-term, K) and carbamidomethyl were set as fixed modifications. Quantifications of spectra were rejected if average S/N values were below 5 across all channels and/or isolation interference exceeded 50%. Protein abundances were calculated by summing all peptide quantifications for each protein.

Reactome analysis were performed with PANTHER using the “Statistical overrepresentation test” tool with default settings. Proteins were considered significantly downregulated for reactome analysis when: FC < −0.5 and *p* < 0.05. Heatmap visualization was performed using Morpheus (Broad Institute).

#### Analysis of publicly available data

All publicly available data and software used for this publication are listed (Appendix Table S5). Oncoprints were generated using cBioportal [63, 64]. Briefly, Oncoprints generates graphical representations of genomic alterations, somatic mutations, copy number alterations and mRNA expression changes. TCGA data was used for the different analysis. Data were obtained using UCSC Xena (10.1101/326470). Data was download as log2(norm_count+1)

Box plots using TCGA and GTEx data were generated using the online tool BoxPlotR [65] and GEPIA [66]. For BoxplotR, the data previously download from UCSC Xena was used to generate the graphics, *p* values were calculated using two-tailed *t* test. For Gepia. The differential analysis was based on: “TCGA tumors vs (TCGA normal)”, whereas the expression data were log2(TPM + 1) transformed and the log2FC was defined as median(tumor) – median(normal). *p* values were calculated with a one-way ANOVA comparing tumor with normal tissue. Tukey and Altman whiskers where used depending of the number of samples. Correlation analysis were calculated using using GEPIA’s software. The analysis was based on the expression of the following datasets: “TCGA tumors”, “TCGA normal”. *p* values for correlation coefficents were calculated using two-tailed Student’s *t* tests.

Heatmap Genomic signature comparing primary human lung tumor samples was performed using UCSC Xena (10.1101/326470) based on the dataset “TCGA tumors”. Compared Gene Expression across different cell lines was perfomed using the online tool R2.

## Supplementary information

Supplementary Figures 1-11

## Data Availability

RNA-sequencing data is available at the Gene Expression Omnibus under the accession number GEO: GSE129982.

## Materials availability

Further information and requests for resources and reagents should be directed to and will be fulfilled by the Lead Contact, Markus E. Diefenbacher (markus.diefenbacher@uni-wuerzburg.de).
