# Supplementary material for: Inhibition of USP28 overcomes Cisplatin-resistance of squamous tumors by suppression of the Fanconi anemia pathway
Source: Cell Death Differ. 2021 Oct 5;29(3):568–84. doi: 10.1038/s41418-021-00875-z (PMC8901929; doi:10.1038/s41418-021-00875-z)
Supplement: Supplementary file 1 — Supplementary Figures 1-11 [file 41418_2021_875_MOESM1_ESM.pdf]

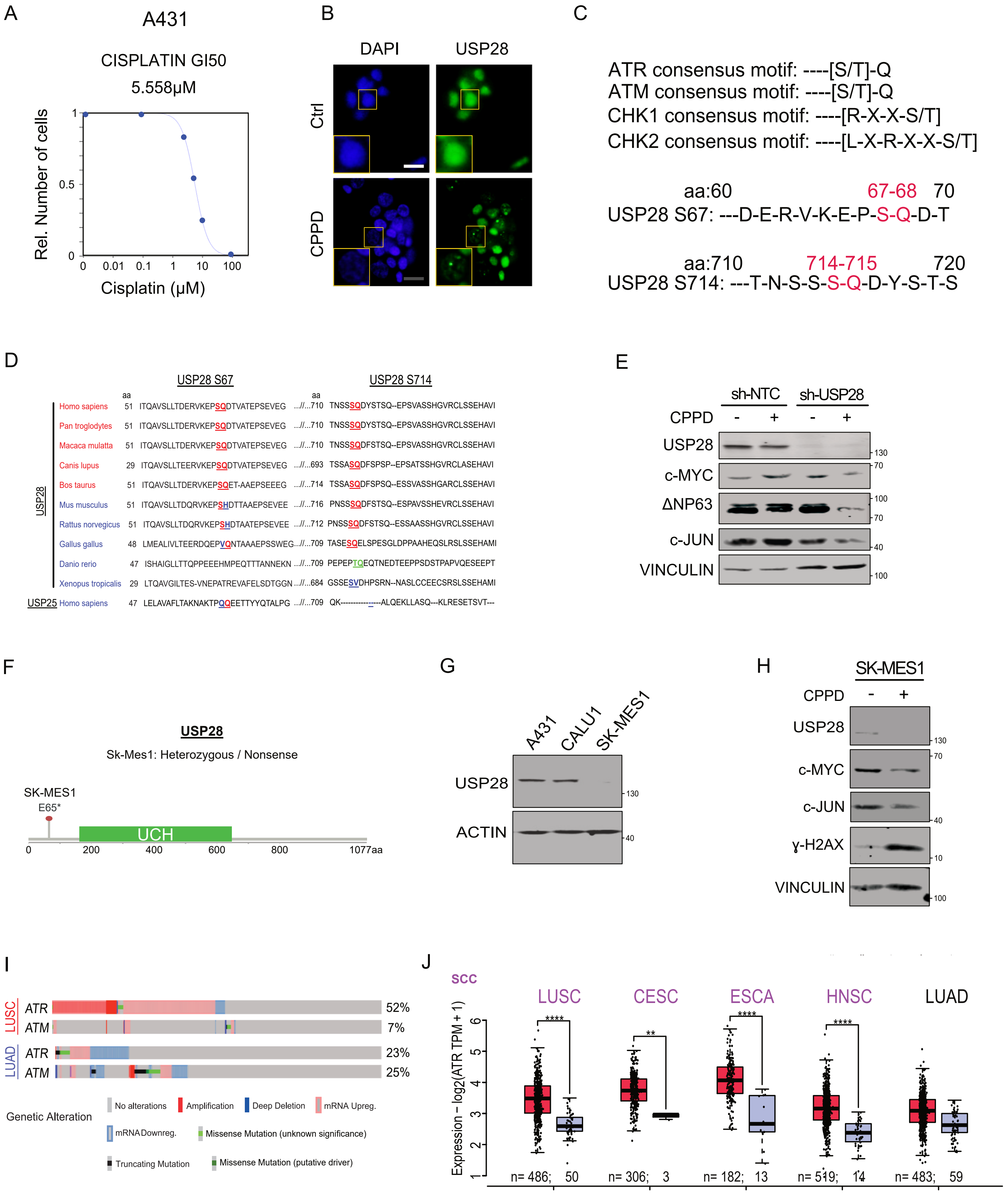

Prieto-Garcia et al. Sup. Figure 1

Figure S1 USP28 is recruited to DNA damage sites and phosphorylated by ATR upon Cisplatin treatment

A) A431 cells were seeded at equal density and co-cultured in the presence of either DMF or 0.1 µM, 2.5 µM, 5 µM, 10 µM or 100 µM CPPD for 48h. Cells were quantified using Casy® cell counter upon exposure to CPPD. 50% growth inhibition (GI_{50}) was calculated from n=3 experiments.

B) Immunofluorescence staining of endogenous USP28 in A431 cells exposed to either DMF or 5 µM Cisplatin for 6 hours. DAPI as nuclear counterstain. Scale bar= 40µm. n=3

C) Schematic representation of the human USP28 sequence. Human USP28 harbours conserved ATM/ATR S/T-Q phospho-motifs at serine 67 and serine 714. USP28 sequence information obtained from www.phosphosite.org.

D) Sequence alignment within USP28 at serine 67 and serine 714 depicting inter-species conservation. Red=Conserved ATM/ATR phospho-motif respect to human; Blue=Non-conserved ATM/ATR phospho-motif with respect to human; Green =SQ ATM/ATR phospho-motif was replaced for TQ ATM/ATR phospho-motif. Data obtained from website: www.ebi.ac.uk.

E) Immunoblot against endogenous USP28, c-MYC, ∆NP63 and c-JUN in A-431 cells, lentivirally transduced with shRNA targeting either a non-targeting control (sh-NTC) or USP28 (sh-USP28), followed by treatment with either DMF or 5 µM CPPD for 6 hours. VINCULIN serves as loading control. n=3.

F) Schematic representation of USP28 mutations present in the SK-MES1 lung cancer cell line. Data obtained from the website cancer.sanger.ac.uk .

G) Immunoblot against endogenous USP28 in the SCC cell lines A431, CALU1 and SK-MES1. ACTIN serves as loading control. Representative immunoblot of n=3 experiments.

H) Immunoblot against endogenous USP28, c-MYC, c-JUN and ɣ-H2AX in the USP28 mutant cell line SK-MES1, treated with either DMF or 5 µM CPPD for 6 hours. VINCULIN serves as loading control. Representative immunoblot of n=3 experiments.

I) Genetic alteration of ATR and ATM in lung SCC (LUSC) and lung ADC (LUAD) tumors. Publicly available patient data obtained from CBIOPORTAL (www.cbioportal.org)

J) ATR gene expression in tumour (red) and normal (blue) samples for lung SCC (LUSC), cervix SCC (CESC), esophagus SCC (ESCA), head and neck SCC (HNSC) and lung ADC (LUAD) patient samples. ** $p<0.01$; **** $p<0.0001$. Data obtained from GEPIA2 online software.

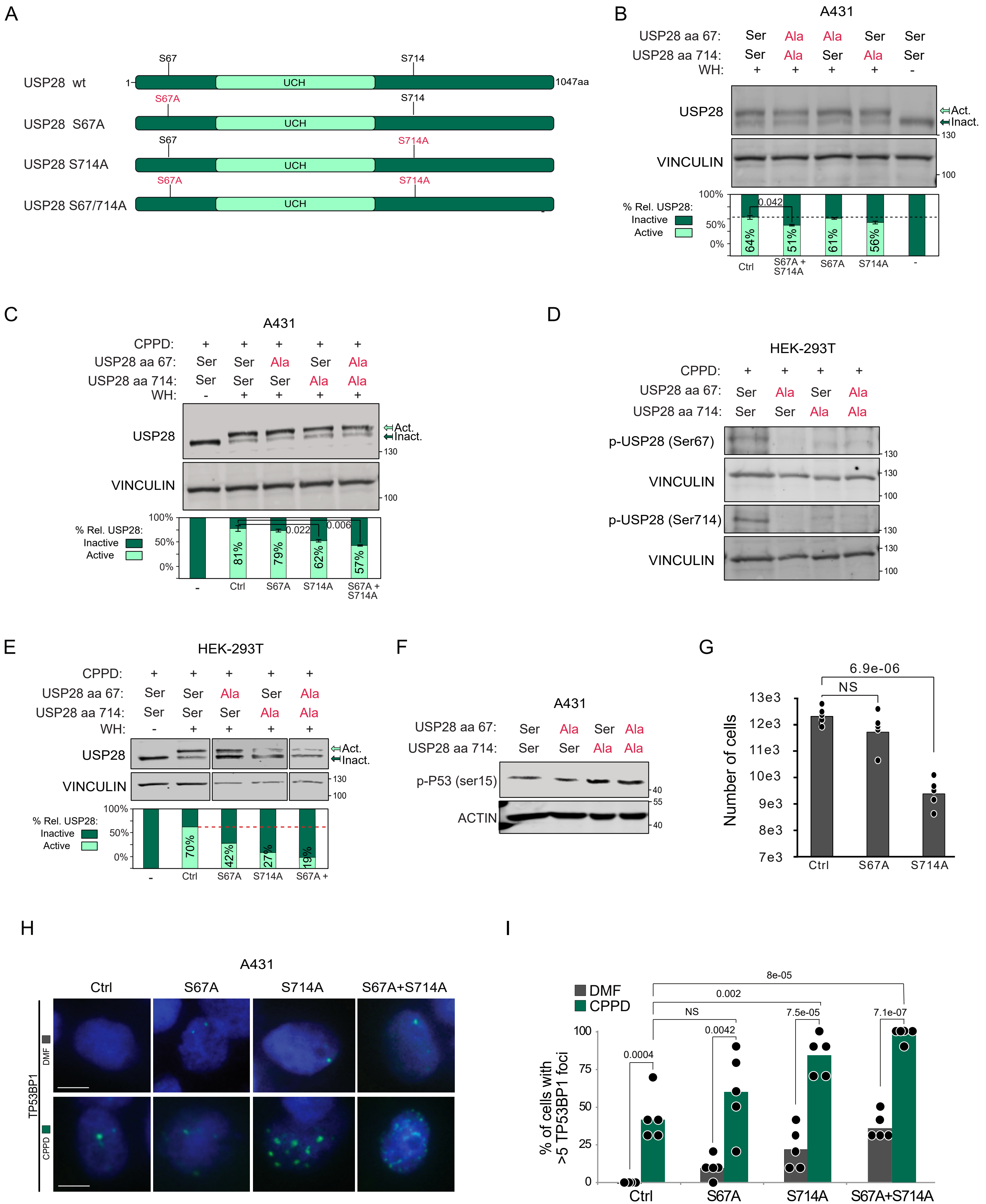

Prieto-Garcia et al. Sup. Figure 2

Figure S2 Phosphorylation of USP28 upon Cisplatin exposure is required to repair DNA damage in SCC

A) Schematic representation of the point mutations (Serine 67 to Alanine and/or Serine 714 to Alanine) introduced into USP28 in A431 and HEK-293T cell lines. Red=mutation; Black=WT

B) Ubiquitin suicide probe (warhead) assay, followed by immunoblotting against USP28 in control, S67A, S714A and S67A+S714A mutant A431 cells. 'Act.' arrow indicates active USP28. 'Inact.' arrow indicates inactive USP28. VINCULIN serves as loading control. Bar graph shows quantification of active and inactive USP28 upon VINCULIN normalization. Quantitative graphic is represented as mean and standard deviation (SD) of three independent biological replicates (n=3). p-values were calculated using two-tailed t-test statistical analysis.

C) Ubiquitin suicide probe (warhead) assay, followed by immunoblotting against USP28 in control, S67A, S714A and S67A+S714A mutant A431 cells exposed to 5 µM CPPD for 6 hours. 'Act.' arrow indicates active USP28. 'Inact.' arrow indicates inactive USP28. VINCULIN serves as loading control. Bar graph shows quantification of active and inactive USP28 upon VINCULIN normalization. Quantitative graphic is represented as mean and standard deviation (SD) of three independent biological replicates (n=3). p-values were calculated using two-tailed T-test statistical analysis.

D) Immunoblotting of phosphorylated USP28 at serine 67 and 714 in control, S67A, S714A and S67A+S714A mutant HEK-293T cells exposed to indicated concentrations of CPPD for 6 hours. VINCULIN serves as loading control. Representative immunoblots of n=3.

E) Ubiquitin suicide probe (warhead) assay, followed by immunoblotting against USP28 in control, S67A, S714A and S67A+S714A mutant HEK-293T cells exposed to 5 µM CPPD for 6 hours. 'Act.' arrow indicates active USP28. 'Inact.' arrow indicates inactive USP28. VINCULIN serves as loading control. Bar graph shows quantification of active and inactive USP28 upon VINCULIN normalization. Quantitative graphic is represented as mean and standard deviation (SD) of three independent biological replicates (n=3). p-values were calculated using two-tailed T-test statistical analysis.

F) Immunoblotting against phosho-TP53 at serine 15 in control, S67A, S714A and S67A+S714A mutant A431 cells treated with 5µM CPPD for 6 hours. ACTIN serves as loading control. Representative immunoblots of n=3.

G) Number of cells in control S67A and S714A mutant A431 cells, treated with DMF for 48 hours. Number of cells were calculated by measuring DAPI positive cells in 15 fields of view per well, n=5 wells per condition. Quantitative graphic is represented as mean of five independent biological replicates (red dots). p-values were calculated using two-tailed T-test statistical analysis.

H) Immunofluorescence against endogenous TP53BP1 (green) in control, S67A, S714A and S67A+S714A mutant A431 cells, treated with either DMF or 5 µM CPPD for 6 hours. Scale bar= 5µm. DAPI served as nuclear marker. Representative images of five independent biological replicates (n=5).

I) Quantification of TP53BP1 positive cells from H). Percentage of cells with more than 5 TP53BP1 foci was calculated measuring 10 cells per field of view, n=5 fields from 5 different wells per condition. Scale bar= 10µm. Quantitative graph is represented as mean of 50 cells (red dots) from five independent wells (n=5). p-values were calculated using two-tailed T-test statistical analysis.

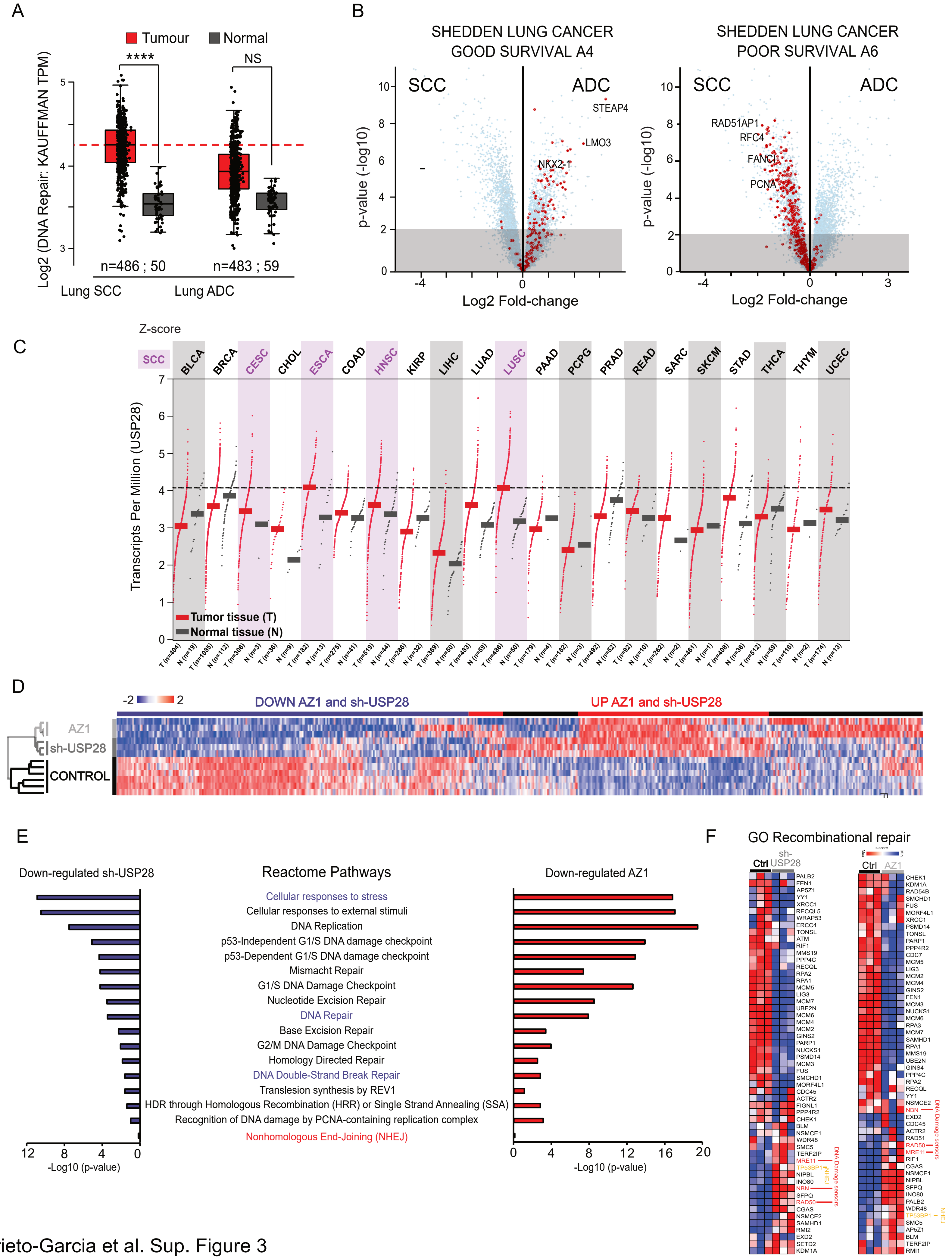

Prieto-Garcia et al. Sup. Figure 3

Figure S3 Loss of USP28 negatively affects the expression of DDR effector proteins in SCC

A) Expression of DNA damage genes according to the described Kauffmann signature in human lung squamous cell carcinomas (SCC, n=486), adenocarcinomas (ADC, n=483) and normal, non-transformed tissue (normal SCC=50, normal ADC=59). Generated with the open source tool www.gepia2.cancer-pku.cn. In box plots, the center line reflects the median, the upper and lower box limits indicates the first and third quartile. Whiskers extend 1.5x the IQR and outliers are marked as dots.

B) Volcano Plots showing the expression of gene signatures according to Shedden lung cancer poor survival A4 (red dots, rigth panel) and Shedden lung cancer good survival A6 (red dots, left panel), comparing lung ADC and SCC patients. Generated with the online tools www.r2.amc.nl.

C) Analysis of public available expression data of USP28 in normal (N) and tumor tissue (T) in a pan-cancer panel. Esophageal (ESCA) and lung squamous cell carcinoma (LUSC) showed highest relative expression of USP28. Squamous tumors are highlighted in pink. Data obtained via the online tool http://gepia2.cancer-pku.cn/

D) Heatmap analysis showing protein changes in the 4503 proteins analysed upon whole cell mass spectrometry. The proteome was analysed in A431 cells upon exposure to AZ-1 (15 µM, 48 hours) or DMSO (control) and in A-431 cells lentivirally transduced with inducible shRNA targeting USP28 (sh-USP28) or non-targeting control (Control). Sh-USP28 and sh-NTC cells were exposed to 1µg/ml Doxycycline for 72 hours prior to analysis Blue=Down-regulated in AZ1/sh-USP28; Red=Up-regulated in AZ1/sh-USP28. n=3.

E) Reactome pathway analysis of proteomic data upon knock down by shRNA or pharmacological targeting of USP28 by AZ-1 in A431 cells. Sh-USP28 and sh-NTC cells were exposed to 1µg/ml Doxycycline for 72 hours prior to analysis. A431 cells were exposed to 15 µM AZ1 or DMSO for 48 hours prior to analysis. Highlighted are pathways involved in DNA damage signalling, response and clearance. Generated with the open source tool www.pantherdb.org. NHEJ (red) was not affected upon AZ-1 nor USP28 genetic depletion. n=3

F) Heatmap analysis according to the GO Recombinational Repair protein signature in control (ctrl) vs sh-USP28 and cells exposed to DMSO (ctrl) vs 15 µM AZ1 A431 cells for 48 hours. Sh-USP28 and sh-NTC cells were exposed to 1µg/ml Doxycycline for 72 hours prior to analysis. Red = DNA damage sensors; Yellow = DNA damage sensor for NHEJ. n=3

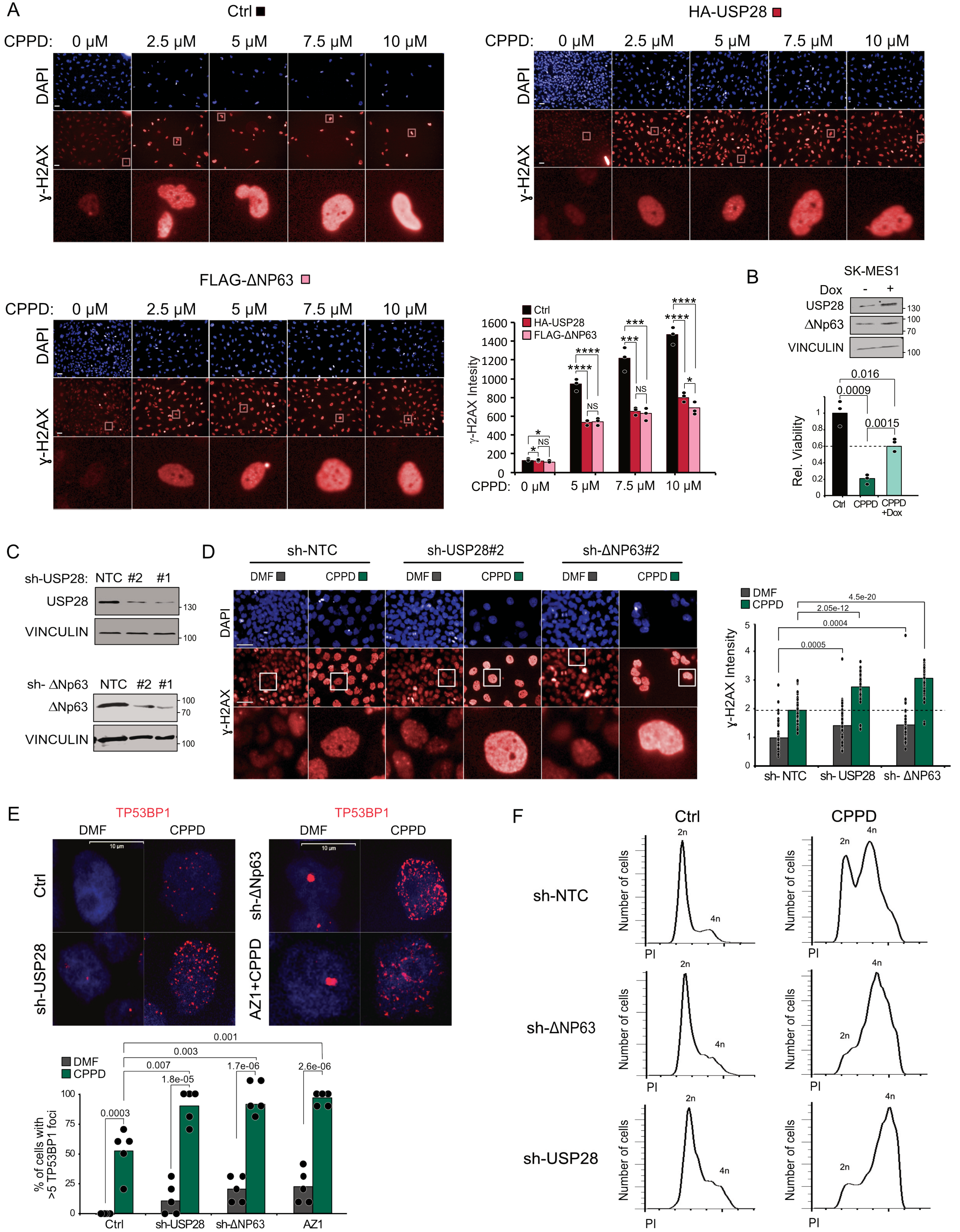

Prieto-Garcia et al. Sup.Figure 4

Figure S4 USP28-ΔNp63 axis is required for DDR upon cisplatin treatment and chemoresistance in SCC

A) Immunofluorescence staining against the DNA damage marker γ-H2AX in BEAS-2B cells transiently transfected with either human USP28 or ΔNP63. Transfection of a GFP cDNA expressing plasmid served as control (-). Cells were exposed to indicated concentrations of CPPD for 48 hours. DAPI served as nuclear marker. Bar graph shows quantification of relative γ-H2AX fluorescence intensity was performed by quantifying 15 fields per well, n=3 wells per condition, via the Operetta High Content Screening microscope. Quantitative graph is represented as mean of n=3 (red dots). Scale bar= 100µm. Pink boxes indicate highlighted cells. p-values were calculated using two-tailed T-test statistical analysis. * = p< 0.05; *** = p<0.001; **** = p<0.0001

B) Immunoblot of USP28 and ΔNP63 in SK-MES1 cells lentivirally transduced with an inducible murine USP28 cDNA (mUSP28), followed by exposure to either DMSO or 1µM DOX for 96 hours. VINCULIN serves as loading control. Relative cell viability was quantified using SK-MES1 cells lentivirally transduced with inducible mUSP28 upon exposure to DMSO, 15µM CPPD or 1µM DOX + 15µM CPPD for 96 hours. Quantitative graph is represented as mean of n=3 (red dots). p-values were calculated using two-tailed T-test statistical analysis. n=3.

C) Immunoblot of USP28 and ΔNP63 in A431 cells lentivirally transduced with two shRNA targeting either USP28 (sh-RNA USP28#1 #2) or ΔNp63 (sh-RNA ΔNp63#1 and #2). VINCULIN serves as loading control. n=3.

D) Immunofluorescence staining against γ-H2AX in lentivirally transduced A431 cells (shRNA-control, sh-RNA USP28#2 or ΔNP63#2) upon exposure to either DMF or 5µM CPPD for 48 hours. DAPI served as nuclear marker. n=3. Quantification of relative γ-H2AX fluorescence intensity in A431 cells. Scale bar= 200 µm, n=50 cells. Two tailed T-Test was used to calculate the p-value.

E) Immunofluorescence staining against endogenous TP53BP1 (red) in A431 cells, lentivirally transduced (shRNA-control, shRNA USP28#1 or ΔNP63#1) and treated with DMF+DMSO (Ctrl) or 5µM CPPD for 48 hours. Alternatively to genetic knock down of USP28 by shRNA, A431 cells were exposed to either 15 µM AZ1 or co-treatment with 5 µM CPPD + 15 µM AZ1 for 48 hours. DAPI served as nuclear marker. Percentage of cells with more than 20 TP53BP1 foci was calculated measuring 10 cells per field of view, n=5 fields from 5 different wells per condition. Quantitative graph is represented as mean of 50 cells (red dots) from five independent wells (n=5). Scale bar= 10µm. p-values were calculated using two-tailed T-test statistical analysis.

F) Propidium iodide FACS-based cell cycle analysis of lentivirally transduced A431 cells (shRNA-control, shRNA USP28#1 or ΔNP63#1) upon exposure to either DMF or 5 µM CPPD for 48 hours. Representative profile of n=3.

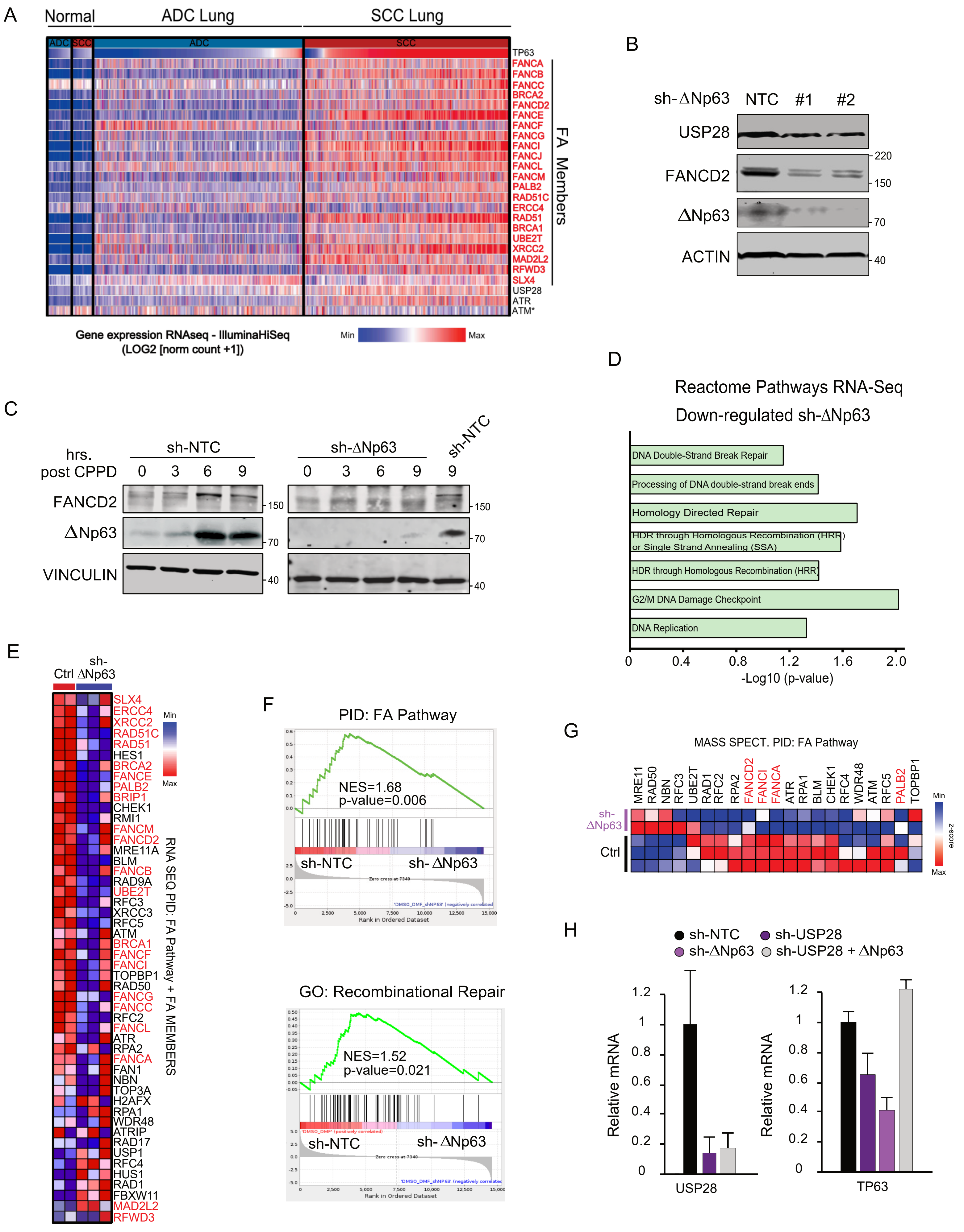

Prieto-Garcia et al. Sup. Figure 5

Figure S5 Deregulation of ΔNp63 impairs the Fanconi Anemia pathway in SCC

A) Publicly available gene expression analysis of TP63, Fanconi Anemia pathway genes, USP28, ATM and ATR in human non-transformed lung tissue, lung ADC and lung SCC. Generated with the online tool www.xena.ucsc.edu. Direct FA key members are highlighted in red.

B) Immunoblot of endogenous USP28, FANCD2 and ΔNP63 in A431 cells lentivirally transduced with inducible shRNA expressing either control or two independent sequences targeting ΔNP63. Cells were exposed to 1µg/ml Doxycycline for 72 hours prior to analysis. ACTIN serves as loading control. Representative immunoblot of n=3.

C) CPPD pulse chase experiment (5 µM CPPD for 1 hour, followed by wash out) in A431 cells lentivirally transduced with control (sh-NTC) or sh-ΔNP63#1, followed by immunoblotting of endogenous FANCD2 and ΔNP63. Numbers indicate hours post CPPD treatment. VINCULIN served as loading control. Representative immunoblot of n=3.

D) Reactome analysis of down-regulated pathways related to DNA damage signalling upon shRNA mediated knock down of ΔNP63 in A431 cells. Generated with the open source tool www.pantherdb.org. The analysis was performed analysing significantly down-regulated genes from RNA-Sequwencing data. Genes were considered significantly downregulated for Reactome analysis when: Log2FC>0.75 and FDR p-value<0.05. Quantitative graphic is represented as -LOG10 p-value of the Reactome pathway indicated.

E) Gene expression Heatmap of FA pathway genes upon RNA sequencing analysis of control and sh-ΔNP63 silenced A431 cells. Direct FA members are highlighted in red. Heatmap was obtained using GSEA software. Minimal gene expression is represented by blue and maximal gene expression by red.

F) Gene set enrichment analysis (GSEA) of FA pathway signature genes and Recombinatorial Repair pathway signature genes, comparing sh-ΔNP63 and sh-NTC A431 cells upon RNA-Sequencing. GSEA analyses were done with signal2Noise metric and 1000 permutations. (*N*)ES: (normalized) enrichment score.

G) Protein abundance Heatmap using Z-scores of FA proteins upon Mass spectrometry comparing control and sh-ΔNP63 knock down A431 cells. Direct FA members are highlighted in red. Heatmap was created using the Broad Institute Morpheus online tool. Minimal row Z-score is represented by blue and maximal row Z-score by red.

H) Quantitative RT-PCR of *USP28 and TP63* relative mRNA expression in A431 cells stably transduced with sh-NTC (grey), sh-USP28 (blue), sh-ΔNp63 (red) or sh-USP28 A431 cells transiently transfected with ΔNp63 (black) to rescue loss of ΔNp63 protein abundance upon knock down of USP28. Values are presented as normalised to ACTB. Quantitative graphic is represented as mean and SD of three independent biological replicates (n= 3).

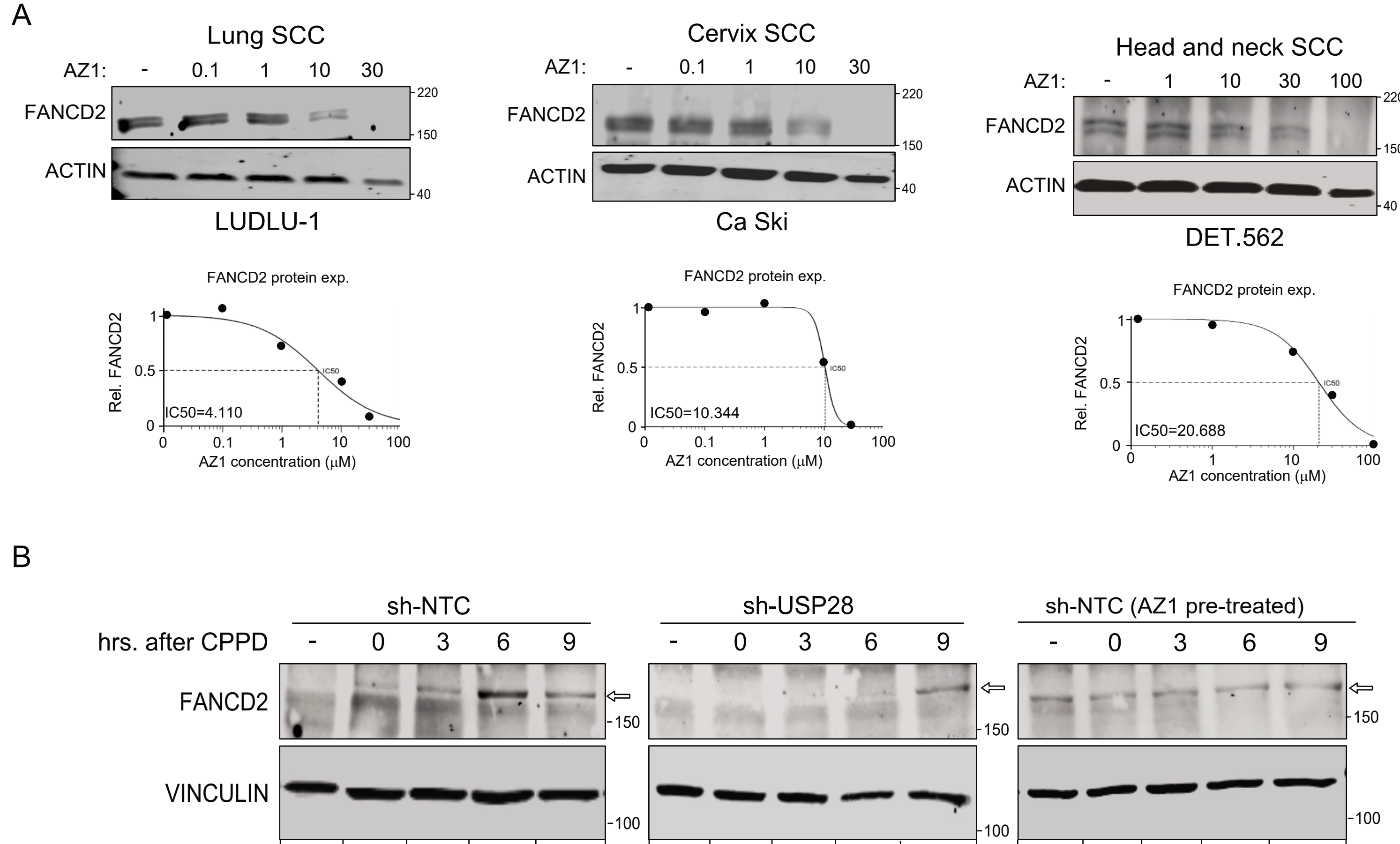

	sh-NTC					sh-USP28					sh-NTC (AZ1 pre-treated)				
hrs. after CPPD	-	0	3	6	9	-	0	3	6	9	-	0	3	6	9
Rel FANCD2:	1	3.3	5.3	18.3	5.8	0.12	0.111	0.25	2.4	5.5	4.6	4.3	4.4	4.2	5.0
SD:	0.3	1.1	0.8	5	1.5	0.1	0.06	0.3	1	0.4	1.7	1	1.8	1.1	0.5
p-value to ctrl:		0.027	0.0009	0.004	0.005	0.008	0.007	0.03	0.04	0.0001	0.023	0.006	0.032	0.009	0.0003

Prieto-Garcia et al. Sup. Figure 6

Figure S6 Pharmacologic inhibition of USP28 via AZ1 affects FANCD2 expression in human lung, cervix and head-and-neck SCC lines

A) Immunoblot of endogenous FANCD2 in the SCC cell lines LUDLU-1 (LSCC), Ca Ski (CESC) and Detroit 562 (HNSCC). Cells were treated for 24 h with either DMSO or indicated concentrations of AZ1. ACTIN served as loading control. FANCD2 half-maximal inhibitory protein abundance (IC_{50}) was calculated. Representative immunoblots of n=3.

B) Immunoblot of FANCD2 in CPPD pulse chase experiment (5 µM, 1 hour exposure, followed by washout) of control (sh-NTC), sh-USP28#1 or sh-NTC+AZ1 in A431 cells. A431 cells were collected at indicated time points after CPPD exposure. Alternatively, sh-NTC cells were cultured in the presence of 15 µM AZ1 at time of 5 µM CPPD and continuously after CPPD washout VINCULIN served as loading control. n=3. 'Arrow' indicated active FANCD2. Representative immunoblots of n=3. Relative amount of protein abundance and standard deviation (SD) are presented from n=3 experiments. Relative protein intensities were quantified respect to sample “sh-NTC -“ (DMF treated sh-NTC A431 cells) upon VINCULIN normalization. pValue calculated using two-tailed student T-test.

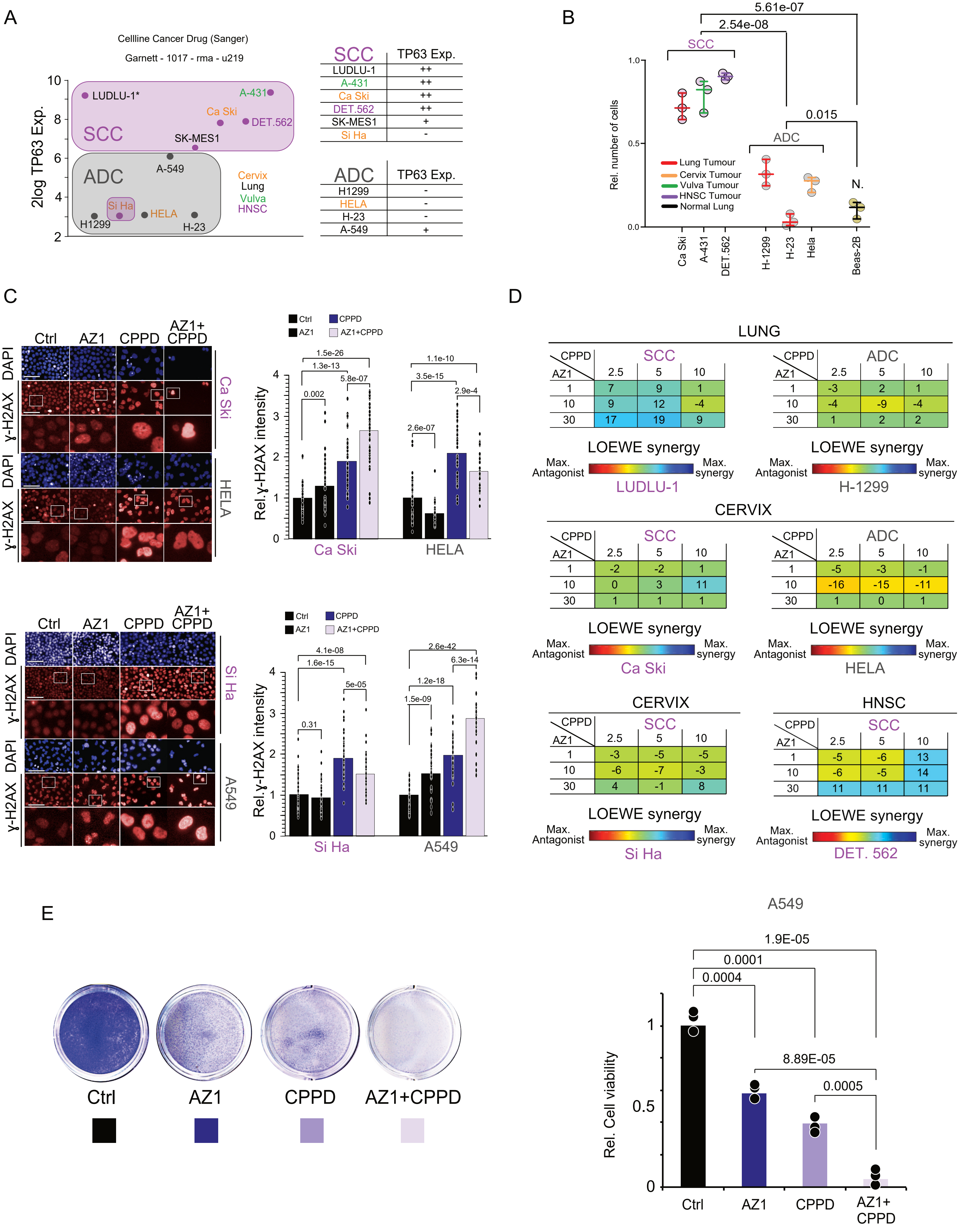

Prieto-Garcia et al. Sup. Figure 7

Figure S7 Pharmacologic inhibition of USP28 re-sensitizes SCC cells to chemotherapy

A) Classification of human cancer cell lines of various origins relative to TP63 expression status. Red box= SCC; Blue box= ADC. The expression of the different cell lines except LUDLU1 (*) were obtained from the dataset: Cellline Cancer Drug (Sanger) Garnett - 1017 - rma - u219. * = LUDLU1 gene expression was obtained from Cell line CCLE Cancer Cell Line Encyclopedia dataset. Data generated with the open source tool www.r2.amc.nl

B) Relative number of A431, Ca Ski, DETROIT 562, H-1299, H-23, HeLa and BEAS-2B cells after exposure to 2.5 µM CPPD for 96 hours. In box plots, the center line reflects the median and the upper and lower limits indicate the first and third quartiles. n=3. p-values were calculated using two-tailed T-test statistical analysis.

C) Immunofluorescence staining of Ca Ski, HeLa, Si Ha and A549 cells treated with DMSO+DMF, AZ1+DMF (Ctrl), CPPD+DMSO or AZ1+CPPD. Cells were treated with 15 µM AZ1, 5 µM CPPD or 15 µM AZ1+5 µM CPPD for 48 hours. DAPI served as nuclear marker. Relative quantification of the ɣ-H2AX staining intensity was measured for the different treatment exposures. Quantitative graphic is represented as median of 50 cells (red dots) from three independent wells. p-values were calculated using two-tailed T-test statistical analysis. Cross indicates mean. n= 50 cells. Scale bar= 200µm. Red= SCC cell line; Blue= ADC cell line.

D) LOEWE synergism score of CPPD and AZ1 in LUDLU-1, H1299, Ca Ski, HeLa, Si Ha and Detroit 562 cell lines. Cells were exposed to indicated concentrations (µM) of CPPD and AZ1 for 48 hours. DAPI was used to assess total cell numbers and quantify LOEWE synergism using Combenefit software. Red= SCC cell line; Blue= ADC cell line.

E) Crystal violet cell viability assay was performed in A549 cells upon treatment with DMSO+DMF (Ctrl), 15 µM AZ1, 5 µM CPPD or 15 µM AZ1+5 µM CDDP for 48 hours. Representative image of n=3. Quantitative graphic is represented as mean of three independent biological replicates (red dots). p-values were calculated using two-tailed T-test statistical analysis.

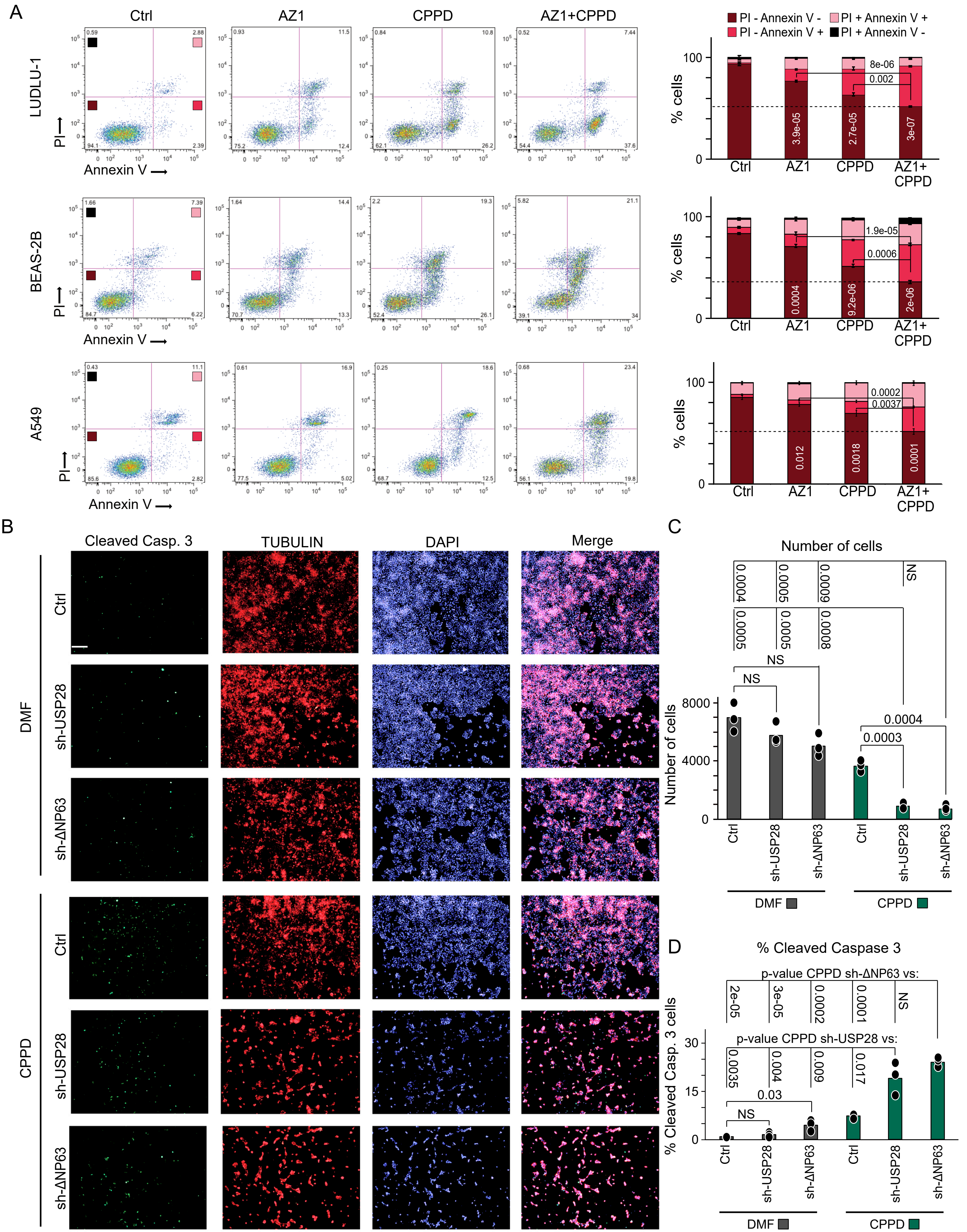

Prieto-Garcia et al. Sup. Figure 8

Figure S8 Pharmacologic Inhibition of USP28 Re-sensitizes SCC Cells to Chemotherapy

A) Propidium Iodide (PI)/Annexin V FACS analysis to assess cell survival and apoptosis of LUDLU-1, BEAS-2B and A549 cells treated with either DMSO/DMF (Control), 15 µM AZ1, 5 µM CPPD or the combination thereof for 48 hours. Bar graph represents the relative amount of PI-/Annexin V- cells, PI-/ Annexin V+ cells, PI+/Annexin V- cells and PI+/Annexin V+ cells. Quantitative graphic is represented as mean and standard deviation (SD) of three independent biological replicates. Representative plot of n=3. p-values were calculated using two-tailed T-test statistical analysis.

B) Immunofluorescence staining of cleaved Caspase 3 (green) TUBULIN (red) and DAPI (blue) of A431 cells lentivirally transduced to express either a non-targeting control shRNA (Ctrl), an shRNA against USP28 (sh-USP28) or ∆NP63 (sh-∆NP63). Cells were exposed to solvent control (DMF) or CPPD (5 µM) for 48 hours. DAPI served as nuclear marker and cell number control. Representative image of n=3

C) Number of sh-NTC, sh-USP28 and sh-∆NP63 A431 cells upon treatment with either DMF or 5µM CPPD for 48 hours. Quantification of DAPI positive cells from B). Quantitative graphic is represented as mean and standard deviation (SD) of three independent biological replicates (red dots). n=3. p-values were calculated using two-tailed T-test statistical analysis.

D) Percentage of cleaved Caspase 3 positive lentivirally transduced sh-NTC, sh-USP28 and sh-∆NP63 cells upon treatment with either DMF or 5µM CPPD for 48 hours. Quantification of cleaved Caspase 3 positive cells from B). Quantitative graphic is represented as mean and standard deviation (SD) of three independent biological replicates (red dots). n=3. p-values were calculated using two-tailed T-test statistical analysis.

A

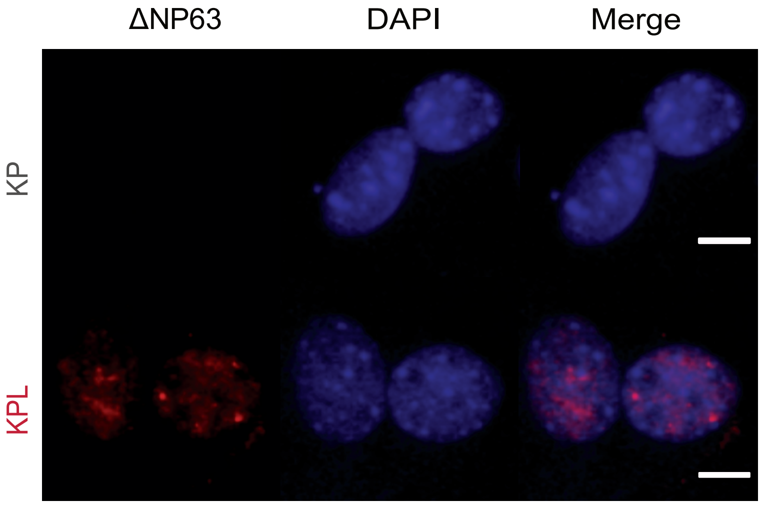

B

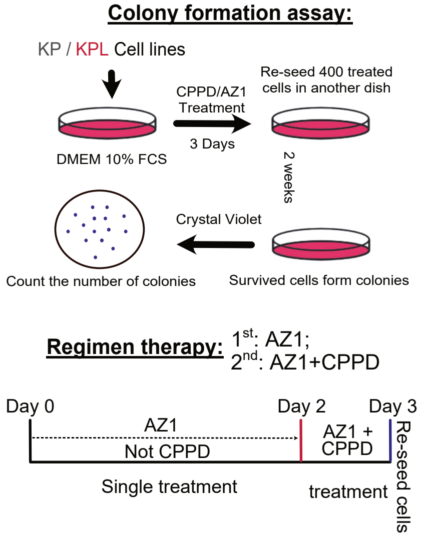

C

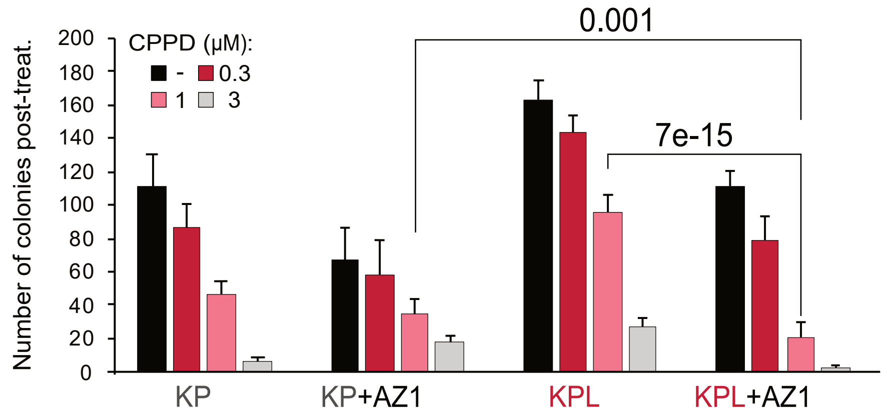

D

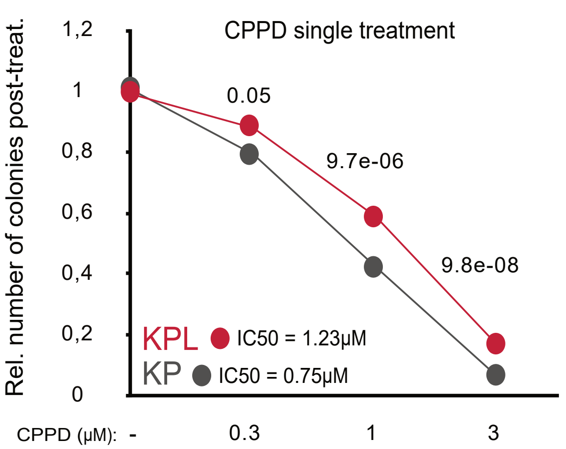

p-values	KP	KP 0.3µM	KP 1µM	KP 3µM	KP +AZ1	KP 0.3µM +AZ1	KP 1µM +AZ1	KP 3µM +AZ1	KPL	KPL 0.3µM	KPL 1µM	KPL 3µM	KPL +AZ1	KPL 0.3µM +AZ1	KPL 1µM +AZ1	KPL 3µM +AZ1
KP		0.023	5e-10	2e-14	2e-05	2e-06	4e-11	2e-13	2e-07	6e-05	0.022	2e-12	NS	0.0002	2e-12	7e-15
KP 0.3µM	0.023		2e-08	9e-15	0.013	0.0008	7e-10	3e-13	2e-12	2e-10	NS	5e-12	8e-05	NS	9e-12	2e-15
KP 1µM	5e-10	2e-08		6-e14	0.003	NS	0.003	1e-10	6e-19	4e-18	5e-12	2e-07	9e-15	6e-07	3e-07	3e-15
KP 3µM	2e-14	9e-15	6e-14		8e-10	3e-08	1e-09	2e-07	7e-23	9e-23	8e-19	3e-11	1e-20	5e-14	8e-05	0.004
KP +AZ1	2e-05	0.01	0.03	8e-10		NS	5e-05	4e-08	2e-12	7e-11	0.0003	9e-07	7e-07	NS	3e-07	2e-10
KP 0.3µM +AZ1	2e-06	0.0008	NS	3e-08	NS		0.002	2e-06	5e-13	1e-11	1e-05	6e-05	5e-08	0.009	1e-05	8e-09
KP 1µM +AZ1	4e-11	7e-10	0.003	1e-09	5e-05	0.002		8e-06	3e-19	2e-18	4e-13	0.012	2e-15	1e-08	0.001	4e-11
KP 3µM +AZ1	2e-13	3e-13	1e-10	2e-07	4e-08	2e-06	8e-06		5e-22	1e-21	3e-17	8e-05	2e-19	2e-12	NS	7e-11
KPL	2e-07	2e-12	6e-19	7e-23	2e-12	5e-13	3e-19	5e-22		0.0005	7e-13	3e-21	1e-12	3e-13	5e-20	2e-23
KPL 0.3µM	6e-05	2e-10	4e-18	9e-23	7e-11	1e-11	2e-18	1e-21	0.0005		1e-10	7e-21	9e-08	2e-11	2e-19	3e-23
KPL 1µM	0.022	NS	5e-12	8e-19	0.0003	1e-05	4e-13	3e-17	7e-13	1e-10		5e-16	0.0008	0.004	7e-15	2e-19
KPL 3µM	2e-12	5e-12	2e-07	3e-11	9e-07	6e-05	0.012	8e-05	3e-21	7e-21	5e-16		3e-18	6e-11	0.05	8e-14
KPL +AZ1	NS	8e-05	9e-15	1e-20	7e-07	5e-08	2e-15	2e-19	1e-12	9e-08	0.0008	3e-18		2e-06	7e-17	2e-21
KPL 0.3µM +AZ1	0.0002	NS	6e-07	5e-14	NS	0.009	1e-08	2e-12	3e-13	2e-11	0.004	6e-11	2e-06		7e-11	1e-14
KPL 1µM +AZ1	2e-12	9e-12	3e-07	8e-05	3e-07	1e-05	0.001	NS	5e-20	2e-19	7e-15	0.05	7e-17	7e-11		2e-06
KPL 3µM +AZ1	7e-15	2e-15	3e-15	0.004	2e-10	8e-09	4e-11	7e-11	2e-23	3e-23	2e-19	8e-14	2e-21	1e-14	2e-06	

0.3, 1 or 3 µM = CPPD concentration AZ1= 15µM **NS**= p-value > 0.05

Prieto-Garcia et al. Figure Sup. 9

Figure S9 Pharmacologic Inhibition of USP28 Re-sensitizes SCC Cells to ChemotherapyA)

A) Immunofluorescence staining of ΔNP63 in KP and KPL mouse cell lines. DAPI served as nuclear marker. Red= SCC cell line; Blue= ADC cell line. Scale bar= 10µm. Cell lines were derived from primary murine NSCLC tumours established by CRISPR mediated genome editing. K= point mutation of the oncogene *Kras* to *KRas*^{G12D}, *P*= truncating mutation in the tumour suppressor *Tp53*, *L* = truncating mutation in the tumour suppressor *Stk11/Lkb1*.

B) Schematic representation of the colony formation assay of the murine ADC cell line KP and the SCC cell line KPL. Cells were either pre-treated with DMSO/DMF (control) or AZ1 for 48 hours, followed by co-exposure to increasing concentrations of CPPD (0µM, 0.3µM, 1µM and 3µM) for another 24 hours. After therapy, 400 treated cells were re-seeded in another dish and maintained in cell culture conditions for two weeks. Finally, formed colonies were stained with Crystal violet stained and quantified.

C) Total number of crystal violet stained colonies upon treatment with either DMSO or 15 µM AZ1 for 48 hours and co-exposure to CPPD or DMF for 24 hours at indicated concentrations in KP (ADC; blue) and KPL (SCC; red) cell lines. The experiment was performed as described in Figure S9B. Quantitative graphic is represented as mean of n=11. p-values were calculated using two-tailed T-test statistical analysis.

D) Relative number of crystal violet stained KP (ADC; blue) and KPL (SCC; red) colonies upon treatment with the indicated concentration of CPPD (0, 0.3, 1 and 3µM) and DMSO for 24 hours. The experiment was performed as described in Figure S9B. Quantitative graphic is represented as mean of n=11. p-values were calculated using two-tailed T-test statistical analysis.

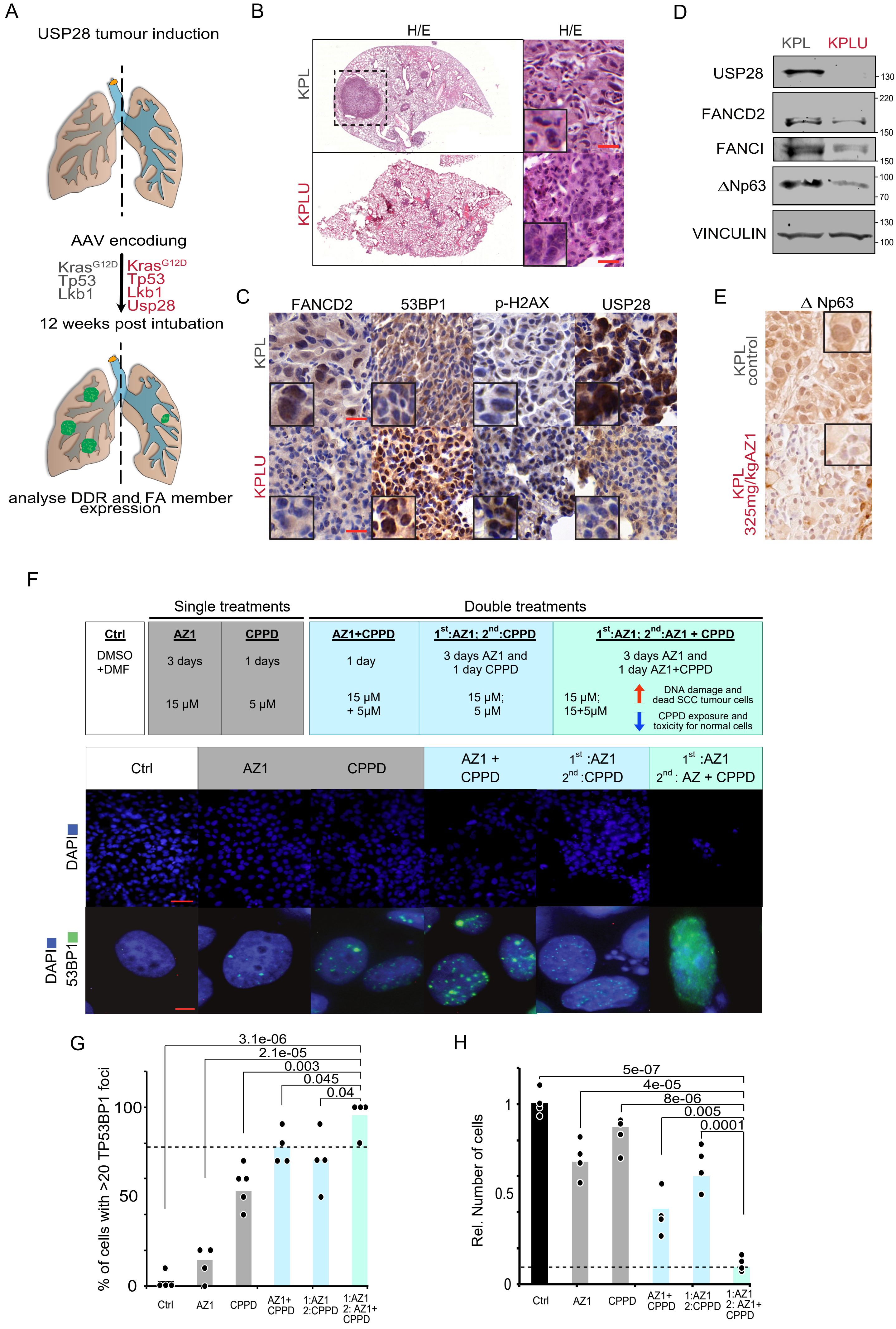

Prieto-Garcia et al. Sup. Figure 10

Figure S10 Inhibition of USP28 activity deregulates FA-DDR signalling in vivo and sensitizes tumours to CPPD treatment in ex vivo organotypic lung SCC tumor slice cultures by de-activating FA

A) Schematic diagram of CRISPR/Cas9-mediated tumour modelling and targeting of *KRas*^{G12D}:*p53*$^{\Delta}$:*Lkb1*$^{\Delta}$ (KPL) or *KRas*^{G12D}:*p53*$^{\Delta}$:*Lkb1*$^{\Delta}$:*Usp28*$^{\Delta}$ (KPLU) in *Rosa26Sor-CAGG-Cas9-IRES-GFP* mice as previously described [35].

B) Representative H&E of lung sections from animals 12 weeks post infection with KPL or KPLU encoding AAV via tracheal instillation as previously described [35]. n=6. Scale bar = 50µm.

C) Representative immunohistochemistry (IHC) staining of FANCD2, TP53BP1, ɣ-H2AX and USP28 in primary KPL and KPLU tumors. n=6. Scale bar = 50µm

D) Immunoblot of endogenous USP28, FANCD2, FANCI and ΔNP63 from KPL or KPLU tumours. VINCULIN served as loading control. Representative immunoblots of three independent biological replicates (n=3)

E) Immunohistochemistry of ΔNP63 in KPL transplant tumors post treatment with either vehicle (PBS/DMSO/Tween) or AZ1 (375mg/kg), for a total of 18 days. n=3. Scale bar = 50µm

F) Upper panel= Schematic representation of treatment strategies combining AZ1 (15 µM) with CPPD (5µM) in A431 cells. Lower panel= Immunofluorescence staining of the DNA damage marker TP53BP1 (green) and DAPI in A431 cells treated with compounds and times according to upper panel, respectively. DAPI serves as nuclear staining control. n=3. Upper scale bar = 100 µm; Lower scale bar= 12 µm.

G) Quantification of TP53BP1 positive cells from F). Percentage of cells with more than 20 TP53BP1 foci was calculated measuring 10 cells per field of view, n=5 fields from 5 different wells per condition. Scale bar= 10µm. Quantitative graph is represented as mean of 50 cells (red dots) from five independent wells (n=5). p-values were calculated using two-tailed T-test statistical analysis.

H) Quantification of relative number of cells from F). Relative surviving number of A431 cells treated according to F) Quantitative graph is represented as mean of four independent wells (red dots). n=4. p-values were calculated using two-tailed T-test statistical analysis.

A

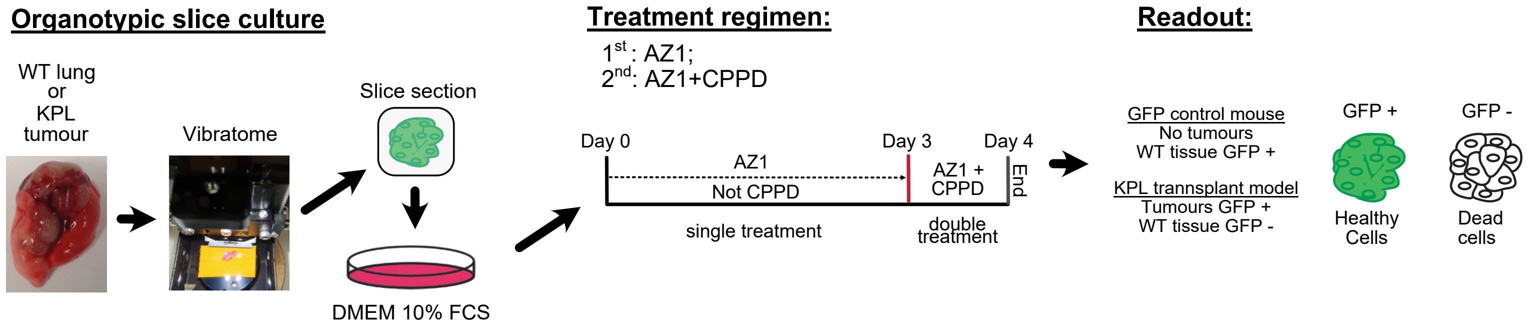

B

Relative GFP organotypic slice culture

p-values	WT	WT 10µM	WT 30µM	WT 100µM	WT +CPPD	WT 10µM +CPPD	WT 30µM +CPPD	WT 100µM +CPPD	Tum.	Tum. 10µM	Tum. 30µM	Tum. 100µM
Tum. +CPPD	**NS**	**NS**	0.042	0.0004	**NS**	**NS**	0.017	0.0003	**NS**	**NS**	0.0017	0.0003
Tum. 10µM +CPPD	**NS**	**NS**	**NS**	0.011	**NS**	**NS**	**NS**	0.007	**NS**	**NS**	0.044	0.01
Tum. 30µM +CPPD	0.0008	0.0017	3e-05	**NS**	0.0001	0.004	0.001	**NS**	7e-06	7e-05	0.004	**NS**
Tum. 100µM +CPPD	0.0004	0.0008	5e-06	0.027	5e-05	0.0016	0.0003	0.048	2e-06	2e-06	0.0002	0.0002

10, 30 or 100 µM = AZ1 concentration CPPD= 10µM **NS**= p-value > 0.05

Prieto-Garcia et al. Sup.Figure 11

Figure S11 Inhibition of USP28 activity deregulates FA-DDR signalling in vivo and sensitizes tumours to CPPD treatment in ex vivo organotypic lung SCC tumor slice cultures by de-activating FA

A) Schematic model of the *ex vivo* organotypic slice culture system (see Material and Methods section for details) and experimental design. Either WT GFP+ lung cells or KPL (p53Δ; Lkb1Δ; KRasG12D) GFP+ lung tumor cells, after orthotopic re-transplantation in wild type C57BL6/J mice, were assessed. The organotypic slice culture was exposed to indicated AZ1 concentrations for 72 hours (0, 10, 30, 100 µM) or DMSO, followed by 24 hours of co-treatment with either solvent control (DMF) or 5µM CPPD. GFP expression was used as a surrogate marker of cell viability.

B) Statistic analysis of Figure 7F, 7G and 7H. Statistic analysis of relative GFP expression of WT GFP+ lung cells or KPL (p53Δ; Lkb1Δ; KRasG12D) GFP+ lung tumor cells, after orthotopic re-transplantation in wild type C57BL6/J mice, within the organotypic slice culture after 72 hours of indicated AZ1 concentrations (0, 10, 30, 100 µM) or DMSO, followed by 24 hours of co-treatment with either solvent control (DMF) or 5µM CPPD. n=4. p-values were calculated using two-tailed T-test statistical analysis.
